# Mesenchymal stem cells in tumor microenvironment: drivers of bladder cancer progression through mitochondrial dynamics and energy production

**DOI:** 10.1038/s41419-024-07068-9

**Published:** 2024-09-20

**Authors:** Enguang Yang, Suoshi Jing, Fang Wang, Hanzhang Wang, Shengjun Fu, Li Yang, Junqiang Tian, Dragan J. Golijanin, Wafik S. El-Deiry, Liang Cheng, Zhiping Wang

**Affiliations:** 1 Institute of Urology, Lanzhou University Second Hospital; Key Laboratory of Gansu Province for Urological Diseases; Gansu Urological Clinical Center, Lanzhou, China; 2 Medical experiment center, Lanzhou University, Lanzhou, China; 3 The Legorreta Cancer Center at Brown University, Department of Pathology and Laboratory Medicine, The Warren Albert Medical School of Brown University, Brown University Health, Providence, RI USA; 4 Division of Urology, Department of Surgery, The Warren Albert Medical School of Brown University, The Miriam Hospital, Providence, RI USA

**Keywords:** Bladder cancer, Cancer microenvironment

## Abstract

Mesenchymal stem cells (MSCs) in tumor microenvironment (TME) are crucial for the initiation, development, and metastasis of cancer. The impact and mechanism of MSCs on bladder cancer are uncertain. Here we analyzed 205 patient samples to explore the relationships between tumor-stroma ratio and clinicopathological features. A co-culture model and nude mouse transplantation were used to explore the biological roles and molecular mechanisms of MSCs on bladder cancer cells. We found that a high tumor-stroma ratio was significantly associated with a larger tumor size and higher T stage, pathological grade, number of vascular invasions, and poor overall survival. MSCs in TME promoted the ability of bladder cancer cells to proliferate, migrate, and invade in vitro and in vivo. Next, we demonstrated that MSCs enhance mitochondrial autophagy and mitochondrial biogenesis of bladder cancer cells, and increase energy production, thereby promoting bladder cancer cell progression. Kynurenine (Kyn) produced by MSCs could enhance mitochondrial function by activating the AMPK pathway. IDO1 inhibitor could reverse the tumor‑promoting effects of MSCs in vitro and in vivo. Our results demonstrated that tryptophan metabolites Kyn of MSCs in TME could enhance mitochondrial function by activating the AMPK pathway, thereby promoting bladder cancer cell progression.

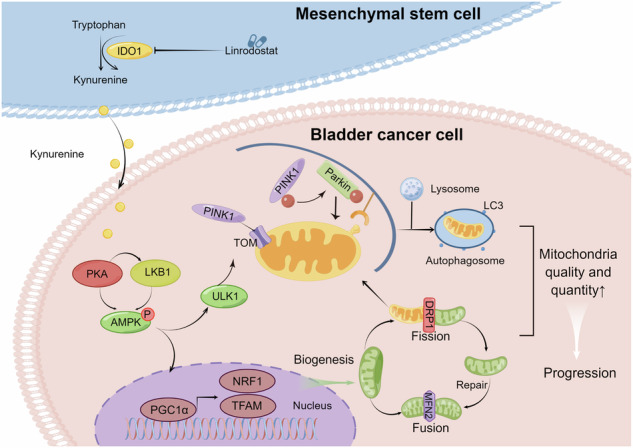

## Introduction

Bladder cancer (BC) ranks as the tenth most prevalent cancer worldwide for both genders, with an estimated 573,000 new cases and 12,000 fatalities in 2020 [1]. Outcomes of patients with BC are poor when it progresses to the muscle-invasive stage [2, 3]. The tumor microenvironment (TME) consists of malignant and non-malignant cells, and current researches concentrate on genetic and epigenetic modifications within the malignant urothelial cells [4, 5]. However, although the stromal cells affect cancer progression and response to therapeutics, they remain relatively understudied [4, 5]. Therefore, it is warranted to explore the impacts of non-malignant cells in TME on BC and to develop a new therapeutic strategy to target these interactions.

Mesenchymal stem cells (MSCs) exhibit the ability to migrate toward tumors in a range of tumor types and exert a consequential influence on the tumor’s course [6, 7] In addition, there is evidence that MSCs exist in the human bladder and urine [8, 9]. While some studies indicate that MSCs can accelerate the growth of tumors, others believe that they have anti-tumor properties [10]. The precise impact and mechanism of MSCs on BC still need more exploration, and novel therapeutic strategies targeting MSCs in the treatment of BC should be developed.

Competition and cross-talk between malignant and non-malignant cells are critical factors in the metabolic reprogramming of tumor cells, which is crucial for the initiation, proliferation, and progression of cancer [11, 12]. The mitochondria served as the center of cellular energy metabolism, exerting a substantial influence on various aspects of cancer, including promotion of tumor anabolism, regulation of redox and calcium homeostasis, participation in transcriptional regulation, and control of cell death, thereby impacting cancer initiation, growth, survival, and metastasis [13, 14]. It is less clear whether MSCs can regulate mitochondrial metabolism to influence the behavior of cancer.

In addition, stromal cells in the TME influence cancer metabolism through local modulation of metabolites, due to unique metabolic profiles, dependencies, and vulnerabilities compared to tumor cells [11]. Although there is mounting evidence indicating that MSCs directly support tumor cells in a direct manner through exosomes or cytokines [10], whether metabolites produced by MSCs influence the behavior of cancer remains uncertain.

In this study, we aimed to investigate the role and contribution of MSCs in the progression of BC. We hypothesized that MSCs promoted BC progression by enhancing mitochondrial function. The findings of this study will provide novel perspectives on the targeted therapy of BC.

## Materials and methods

### Patients and samples

We assess the tumor-stroma ratio score of all BC patients who received transurethral resection of BC or radical cystectomy at the Lanzhou University Second Hospital Department of Urology from May 1, 2017, to December 22, 2019. The computerized medical records were examined to collect clinical data and original pathology reports.

From March 1, 2023, to May 17, 2023, we collected 26 urine samples from 18 BC patients and 8 healthy volunteers, and then detected the level of tryptophan metabolite kynurenine (Kyn). Informed consent was obtained electronically. The study was approved by the Ethics Committee of Lanzhou University Second Hospital (2021A-251, 2023A-261), and was conducted in accordance with the ethical standards of the Declaration of Helsinki.

### Tumor-stroma ratio scoring

The most invasive region of the original tumor was selected for standard microscopy analysis of the tumor-stroma ratio score and T-status. The tumor-stroma ratio was measured as previously mentioned [15]. As established in prior studies on breast cancer, esophageal adenocarcinoma, and colon cancer, a 50% threshold was applied. The classification of tumor-stroma ratio involved distinguishing between stroma low and stroma rich categories.

### Estimation of MSCs infiltration and the calculation of stromal score

The transcript per million formats RNA-seq data and clinical information relevant to Urothelial Bladder Carcinoma (TCGA-BLCA) samples were obtained from TCGA datasets (https://portal.gdc.cancer.gov/). We then screened a total of 405 BC patients from the TCGA-BLCA dataset who had prognostic data available, with follow-up times exceeding 365 days. The Estimation of STromal and Immune cells in MAlignant Tumor tissues using Expression data (ESTIMATE) algorithm was utilized to calculate the stromal score, which indicates the level of stromal infiltration in each tumor sample, through the estimate R package [16]. We estimate the abundance of MSCs in TCGA datasets via the xCell algorithm based on gene signature enrichment [17].

### Flow cytometry analysis and MSCs sorting

To analyze the presence of MSCs in human primary BC tissue, tissue cores from BC specimens were promptly obtained post-surgery. The tissue underwent digestion, leading to a single-cell suspension achieved through a combination of mechanical and collagenase IV methods. The resulting tumor slurry was then transferred to a 15-ml centrifuge tube by passing it through a 100-mesh filter and subsequently centrifuged at 300 × *g* for 5 min. To remove red blood cells, lysis was performed using an ammonium chloride solution (STEMCELL Technologies). Following this, the cells were stained with antibodies in a dark environment for 30 min and subsequently analyzed or sorted using flow cytometry (FCM). The specific antibodies employed were APC/Cyanine7 anti-human CD14(BioLegend, 301820), Alexa Fluor® 700 anti-human CD34(BioLegend, 343526), FITC anti-human CD19(BioLegend, 302206), PE anti-CD105 (BioLegend, 800504), PE/Cyanine7 anti-human CD90 (BioLegend, 328124), PerCP/Cyanine5.5 anti-human CD73(BioLegend, 344014), and APC-CD45(BioLegend, 304012). The analysis was conducted using a FACS Canto flow cytometer (BD Biosciences).

### Tri-lineage differentiation capacity assay of p-MSCs

Cells were cultured until 60–70% confluent in basal media and then induced towards osteogenic, chondrogenic, and adipogenic lineages using a Differentiation Kit (Fuheng Biology, Shanghai, China) according to the manufacturer’s instructions. After 3–4 weeks of induction, osteogenic differentiation was assessed using Alizarin Red staining, chondrogenic differentiation was assessed using Alcian Blue staining, and adipogenic differentiation was assessed using Oil Red O staining.

### Cell lines and cell culture

Two human BC cell lines (UMUC3 and T24) were purchased from Cell Bank, Chinese Academy of Sciences. Human adipose-derived MSCs were purchased from ATCC (American Type Culture Collection). The UMUC-3 and T24 cells were cultured in RPMI-1640 (1640) (Gibico, USA). The MSCs were cultured in specialized media (ATCC, USA). RPMI-1640 was mixed with 1% antibiotics (100 U/ml penicillin and 100 μg/ml streptomycin sulfates) and 10% FBS. The MSCs specialized media contains basal medium (PCS-500-030, ATCC, USA) and Growth Kit (PCS-500-040, ATCC, USA). Then plates were placed in an incubator at 37 °C with an atmosphere of 5% CO_2_.

The transwell chamber (0.4 mm pore size, SPL) was used to establish a co-culture model of MSCs and BC cell lines. MSCs were inoculated into the upper chamber of transwell, while BC cell lines T24 or UMUC-3 were inoculated into the lower chamber. The upper chamber contained an equal 1640 medium as a control. The two chambers of cells were separated by a semi-permeable membrane but shared the same culture medium.

### Generation of conditioned medium

MSCs were plated in 75 cm^2^ flasks. Once reaching 80% confluence, the cells were washed with PBS and re-incubated with 5 ml complete medium at 37 °C with 5% CO_2_. After 24 h, the conditioned medium (CM) was collected, spun to remove cellular debris (1200 rpm for 10 min), and passed through a 0.22 μm filter. Aliquots were frozen and stored at −80 ˚C until use.

### Cell growth and colony formation assays

For CCK-8 assays, T24 and UMUC-3 cells were seeded into 96-well plates at the density of 1 × 10^3^ (cells/well). After 24 h cultivation, different concentrations of MSC-conditioned medium (MSC-CM) or control medium were added respectively. Each well was incubated with 10 μl CCK-8 solution for 2 h away from light before measuring the absorbance at 450 nm by Biotek Multilabel Plate Reader on day 2.

For cell colony formation assays, 500 T24 and UMUC-3 cells were incubated with MSCs in 6-well plates at 37 °C, 5% CO_2_. Two weeks later, the cells were stained with crystal violet (0.2%) for 30 min and the colony numbers were counted.

### 5-Ethynyl-20-deoxyuridine (EdU) incorporation assay

T24 and UMUC-3 cells were seeded at a density of 25 × 10^4^ cells per well in 6-well plates. The newly synthesized DNA of the cells was assessed by the EdU incorporation assays using a Cell-Light EdU DNA Cell Proliferation Kit (Beyotime, China), according to the manufacturer’s instructions. The EdU incorporation rate was expressed as the ratio of EdU-positive cells (green cells) to total Hoechst33342 positive cells (blue cells).

### Cell migration assays

Wound healing assays were employed to assess the migratory potential of cancer cells. Cells were seeded in six-well plates and permitted to reach 100% confluency. Subsequently, the cells were cultured in fresh FBS-free medium, and scratches were created using a 200 μl pipette tip. Images were captured at various time points with an inverted microscope, and the rate of wound closure was quantified using NIH ImageJ software (Version 1.8.0).

### Transwell invasion assay

Invasion assay was performed using the Corning Biocoat Matrigel invasion chambers (Corning 354480) as per manufacturer’s protocol. Briefly, 20,000 BC cell lines T24 or UMUC-3 were inoculated into the upper inserts, and MSCs were inoculated into the lower chamber. After cell adherence, the cancer cells in the upper insert were co-cultured with MSCs in the lower chamber and an equal volume of 1640 medium as the control. After cell migration, the inserts were discarded, and the upper side of the filter was swabbed to remove the nonmigratory cells. The filters were then fixed in 4% paraformaldehyde and stained with 0.5% Crystal violet for 20 min. Microscopic examination was performed and 8 randomly non-overlapping fields were selected to count the stained migrated cells. All the experiments in each group were performed in triplicate.

### Detection of metabolites based on liquid chromatography-mass spectrometry (LC–MS)

The LC-MS/MS system was employed for metabolomic analysis, focusing on the examination of energy metabolites in tumor cell samples co-cultured with MSCs to elucidate the influence of MSCs on tumor cell metabolism. Metabolite profiling in an untargeted manner was carried out on MSC-CM, and the detection of tryptophan metabolites was performed in both MSC-CM and urine samples. Energy metabolites and metabolites of tryptophan were analyzed by MetWare Biotechnology Co., Ltd (Wuhan, China) using an LC-MS/MS system based on an established protocol [18]. For positive ion mode, a 0.1% formic acid aqueous solution and 0.1% formic acid acetonitrile were used. In negative ion mode, water (2 mM ammonium acetate) and acetonitrile were employed. Data acquisition utilized Analyst 1.6.3 software (Sciex), and Multiquant 3.0.3 software (Sciex) was employed for the quantification of all metabolites. Principal component analysis (PCA) conducted with the prcomp function in *R* unveiled significantly regulated metabolites between groups, employing variable importance in projection (VIP) ≥ 1 and absolute Log2FC (fold change) ≥1.0 (fold change ≥ 2). VIP values, extracted from orthogonal partial least-squares discriminant analysis (OPLS-DA) results, which encompass score plots and permutation plots, were generated using the R package MetaboAnalystR.

### Proteomic analysis based on LC–MS/MS

Cellular proteins underwent digestion, reduction, alkylation, solubilization, and quantification following established protocols [19]. Peptide samples totaling approximately 2 µg (5 μL) were introduced into a Thermo Scientific Q Exactive PLUS, coupled online to an EASY-nLC 1200 system, operating in data-independent acquisition (DIA) mode. The liquid chromatography employed buffer A1 (0.1% formic acid) and buffer B1 (80% acetonitrile, 0.1% formic acid) as mobile phases. Peptide separation occurred at a constant flow rate of 300 nL/min, featuring a 160 min non-linear gradient: buffer B1 (5–35%) for 85 min, buffer B1 (35–100%) for 60 min, and buffer B1 (100%) for 15 min. Raw data underwent analysis against the UniprotKB reference proteome of Homo sapiens (as of May 3rd, 2020) using DIA-NN [20]. All parameters were configured with default values, and protein and peptide false discovery rates were established at 0.01.

### Mitochondrial membrane potential assay

A mitochondrial membrane potential (MMP) assay kit with Mito-Tracker Red CMXRos (C1035; Beyotime) was used to determine the MMP. According to the manufacturer’s protocols, cells were incubated with 200 nM Mito-Tracker Red CMXRos for 20 min in the dark at 37 °C. Next, the cells were washed twice with PBS and detected with a fluorescence microscope or flow cytometry.

### Seahorse XF cell mito stress analysis

XF Cell Mito Stress Test Kit was used to measure the mitochondrial respiratory capacity (Agilent Technologies, 103015–100). Cells were seeded in the XF24 Cell Culture Microplate at a density of 1 × 10^4^ cells per well. Microplate was incubated for 24 h at 37 °C with regular medium (control group) or MSC-CM (MSC group). The utility plate with 200 m l of Seahorse Calibrant was used to hydrate the Seahorse XF24 FluxPak sensor cartridge overnight in a non-CO_2_ incubator at 37 °C. The following day, cells were cultured for 1 h in the base medium before being subjected to the experiment, which contains 2 mM L-glutamine, 1 mM sodium pyruvate, and 10 mM glucose. By using the XFe24 extracellular flux analyzer with consecutive injections of 1 μM oligomycin A, 1 μM FCCP, and 0.5 μM rotenone/antimycin A, the oxygen consumption rate (OCR) was determined. Following the experiment, cell lysates were extracted using 20 μl of western blot lysis solution, and the protein concentration was determined using a BCA Protein Assay Kit. The protein concentration in each well was used to standardize the OCR value.

### Transmission electron microscopy analysis

Cells were fixed with 3% glutaraldehyde for 1 h at 4 °C, and then postfixed with 1% osmium tetroxide for 1 h at 4 °C. Samples were dehydrated through a graded series of acetone, infiltrated and then embedded in epoxy embedding medium (Epon812). After being stained with uranyl acetate and lead citrate, cell samples were analyzed with a JEM-1400 FLUSH transmission electron microscope (TEM). All steps were performed according to the manufacturer’s instructions.

### Immunofluorescence staining

The paraffin-embedded tissue sections were deparaffinized by immersing them in xylene for 10 min twice. Gradually rehydrate the sections by passing them through a series of graded ethanol solutions (100%, 95%, 85%, and 75%) for 3 min each. Then the slides were immersed and heated in antigen retrieval buffer (P0088, Beyotime, China) in a microwave according to the manufacturer’s instructions. After cooling down to room temperature, the sections were incubated in a blocking solution (P0102, Beyotime, China) for 1 h at room temperature. Then, incubate the primary antibody overnight at 4 °C. Incubatefluorescent-labeled secondary antibody for 1 h at room temperature in the dark. Stain the nuclei with DAPI (C1005, Beyotime, China). Visualize the immunofluorescent staining using a fluorescence microscope.

### RNA Interference

To knock down the expression of IDO1, RNA interference was performed as follows. The MSC cells were transfected with IDO1 siRNA using Lipofectamine 2000 (Invitrogen, 1803709, Waltham, MA, USA) for 24 h according to instructions. The efficiency of gene knockdown was evaluated by Western blot. The siRNA IDO1 was synthesized and purchased from Tsingke Biotechnology (Beijing, China). All siRNA oligonucleotide sequences used are listed in Table S1.

### Animal studies

Female BALB/c nude mice aged 5 weeks were bought from Gempharmatech Co., Ltd. (Jiangsu, China). To make sure the mice were healthy before the in vivo study began, they were kept in a specialized pathogen-free facility and given a week to acclimate. All animals were treated strictly in line with Guide for the Care and Use of Laboratory Animals of the National Institutes of Health. The Institutional Animal Care and Use Committee at Lanzhou University second hospital gave its approval to the protocol (D2022-335, D2023-057). UMUC-3 cells (1 × 10^6^) or UMUC-3 cells (1 × 10^6^) with MSCs (1 × 10^6^) at a 1:1 ratio suspended in 100 ml 1640 were inoculated subcutaneously (s.c.) into the flanks of the mice form of BC. After 10 days, the mice that had developed tumors.

To evaluate the influence of MSC on tumorigenesis and tumor growth, 100 ml PBS was injected to mice in the control group around the tumor. MSCs (1 × 10^6^) in 1640 (100 ml) were injected into the mice in the MSC group around the tumor site. PBS/MSCs were administered every 2 days. To evaluate whether the IDO1 inhibitor linrodostat could reverse the pro-tumorigenic effects of MSCs, PBS or linrodostat (8 mg/kg) was injected intraperitoneally every 2 days. A vernier caliper was used to measure the tumor’s size every 3 days.

### Total protein extraction and Western blot

The cells were washed with PBS and lysed using ice-cold RIPA (R0010, Solarbio, Shanghai, China) and supplemented with the phosphatase inhibitor cocktail (P1045, Beyotime, Shanghai, China) and PMSF (ST506, Beyotime, Shanghai, China). Cell lysates were separated on 7.5%, 10%, or 12% SDS-PAGE gel and electroblotted onto a nitrocellulose membrane following centrifugation and denaturation by boiling. Antibodies against PKA (55382-1-AP, Proteintech), LKB1 (10746-1-AP, Proteintech), AMPKα (ab32047, Abcam), phospho-AMPKα-Thr172 (40H9, CST, Boston, USA), PINK1(23274-1-AP, Proteintech), ACTB (20536-1-AP, Proteintech), PARK2 (14060-1-AP, Proteintech), PGC1α(66369-1-Ig, Proteintech), NRF1(HY-P80769, MCE), TFAM(ab176558, Abcam), DRP1 (12957-1-AP, Proteintech), MFN2 (12186-1-AP, Proteintech), Tublin(66031-1-Ig, Proteintech), GAPDH(60004-1-Ig, Proteintech), IDO1(ab211017, Abcam), E-cadherin(20874-1-AP, Proteintech), N-cadherin(22018-1-AP, Proteintech), and Vimentin (10366-1-AP, Proteintech) were diluted according to instruction, and the nitrocellulose membrane was incubated with these antibodies. Fluorescent secondary antibodies against rabbit or rat (Licor, Lincoln, USA) were used after diluting 10000 and 15000 times, respectively. The analysis was performed using the Odyssey CLX near-infrared dual-color fluorescence imaging system (Licor).

### Statistical analysis

Statistical analyses were performed using GraphPad Prism 8 software. Differences between the two groups were analyzed with the Student’s t test. For others, the significance was examined by two-way analysis of variance (ANOVA). All data were presented as mean ± SD (triplicate determination). And a *P* value < 0.05 was considered statistically significant. *P < 0.05, **P < 0.01, ***P < 0.001, ****P < 0.0001.

## Results

### High tumor-stroma ratio and MSCs infiltrations are adverse factors in the development of bladder cancer

**Fig. 1:**
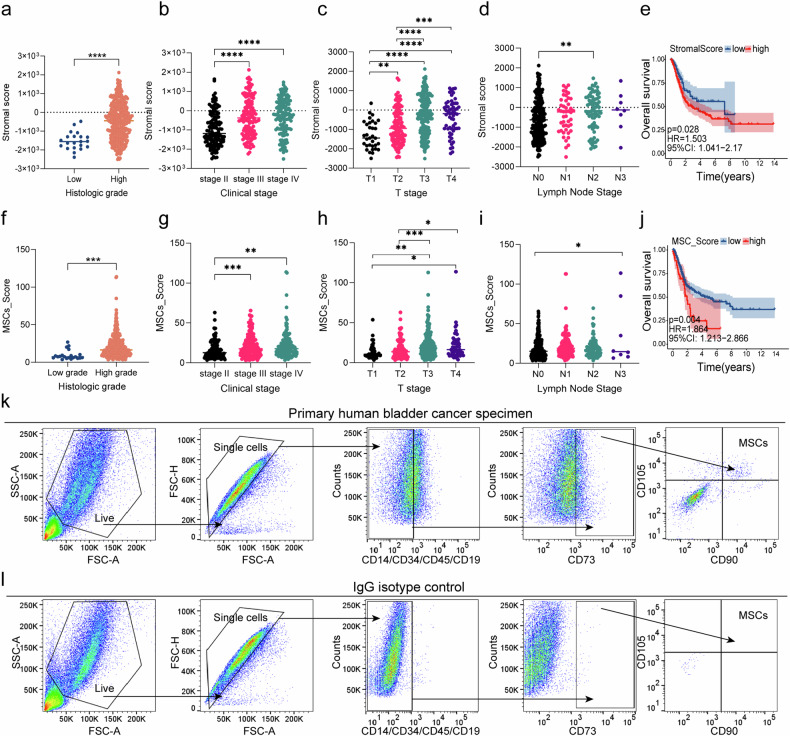
Higher MSCs infiltrations and stromal scores were significantly associated with adverse pathological characteristics and poorer OS in bladder cancer patients. Higher stromal score and MSCs infiltrations was significantly associated with a higher pathological grade (**a**), a higher clinical stage (**b**), a higher T status (**c**), and a higher lymph node stage(**d**). Higher MSCs infiltrations was significantly associated with a higher pathological grade (**f**), a higher clinical stage (**g**), a higher T status (**h**), and a higher lymph node stage (**i**). Kaplan–Meier curves indicated higher stromal scores and MSCs infiltrations were significantly associated with poorer OS in TCGA-BLCA datasets (**e**, **j**). Method for quantifying MSCs in primary human bladder cancer samples (**k**, **l**). *P < 0.05, **P < 0.01, ***P < 0.001, ****P < 0.0001.

205 patients were included in the study, including 56 patients in the stroma-rich group and 149 patients in the stroma-poor group. The patient and pathological characteristics were shown in Table 1. The mean age of the patients was 68 years and 65 years in the stroma-rich and stroma-poor groups, respectively. There were 38 men and 18 women in the stroma-rich group, and 128 men and 21 women in the stroma-poor group. The stroma-rich group exhibited significantly higher mean tumor size, T stages, rate of vascular invasion, and rate of high-level pathological grade compared to the stroma-poor group (*P* < 0.05). Next, we estimated stromal score and abundance of MSCs in TCGA datasets. Higher stromal score and MSCs infiltrations was significantly associated with a higher pathological grade, a higher clinical stage, a higher T status, and a higher lymph node stage (Fig. 1a–d, f–i). The prognostic values on overall survival (OS) were evaluated via log-rank tests. Kaplan–Meier curves indicated that higher MSCs infiltrations and stromal scores were significantly associated with poorer OS in BC patients (*P* < 0.05) (Fig. 1e, j).

**Table 1:** The relationship between tumor-stroma ratio and pathological characteristics.

Characteristic	High, *N* = 56	Low, *N* = 149	*p* value
Sex			0.003
Female	18 (32%)	21 (14%)	
Male	38 (68%)	128 (86%)	
Age (years)	68 (60, 73)	65 (56, 71)	0.3
Tumor size (cm)	3.00 (1.60, 3.95)	2.00 (1.50, 3.00)	0.036
T stage			<0.001
Ta	14 (25%)	86 (58%)	
T1	17 (30%)	22 (15%)	
T2	13 (23%)	26 (17%)	
T3	9 (16%)	7 (4.7%)	
T4	3 (5.4%)	8 (5.4%)	
Local metastasis	4 (7.1%)	9 (6.0%)	0.8
Vascular invasion	14 (25%)	11 (7.4%)	<0.001
Lymph node metastasis	2 (3.6%)	5 (3.4%)	>0.9
Pathological grade			<0.001
High	43 (77%)	67 (45%)	
Low	10 (18%)	80 (54%)	
PUNLMP	3 (5.4%)	2 (1.3%)

In order to verify the presence of MSCs in human primary BC tissue, a flow cytometry-based assay was conducted. The tissue was digested into a single-cell suspension using a combination of mechanical and enzymatic methods. MSCs within this cell population were identified as CD73, CD90, and CD105 triple-positive cells, while lacking CD14, CD34, CD45, and CD19 labeling (Fig. 1k, l). Out of the 12 specimens examined in this study, MSCs accounted for 0.023%~1.490% of the total cell population within the BC tissue (Table 2).

**Table 2:** Quantification of MSC s in primary human bladder cancer samples.

Samples	T stage	Pathological grade	MSCs (%)
BC-1	T1	Low	1.003%
BC-2	T1	High	1.170%
BC-3	T2	High	0.040%
BC-4	T1	Low	1.177%
BC-5	T1	High	0.267%
BC-6	T1	High	0.023%
BC-7	T1	Low	0.213%
BC-8	T1	Low	0.057%
BC-9	T1	Low	1.490%
BC-10	T4	High	0.830%
BC-11	T1	Low	0.177%
BC-12	T1	Low	1.173%

*BC* bladder cancer, *MSC* mesenchymal stem cell.

### MSCs promote progression of bladder cancer cells in vitro and in vivo

**Fig. 2:**
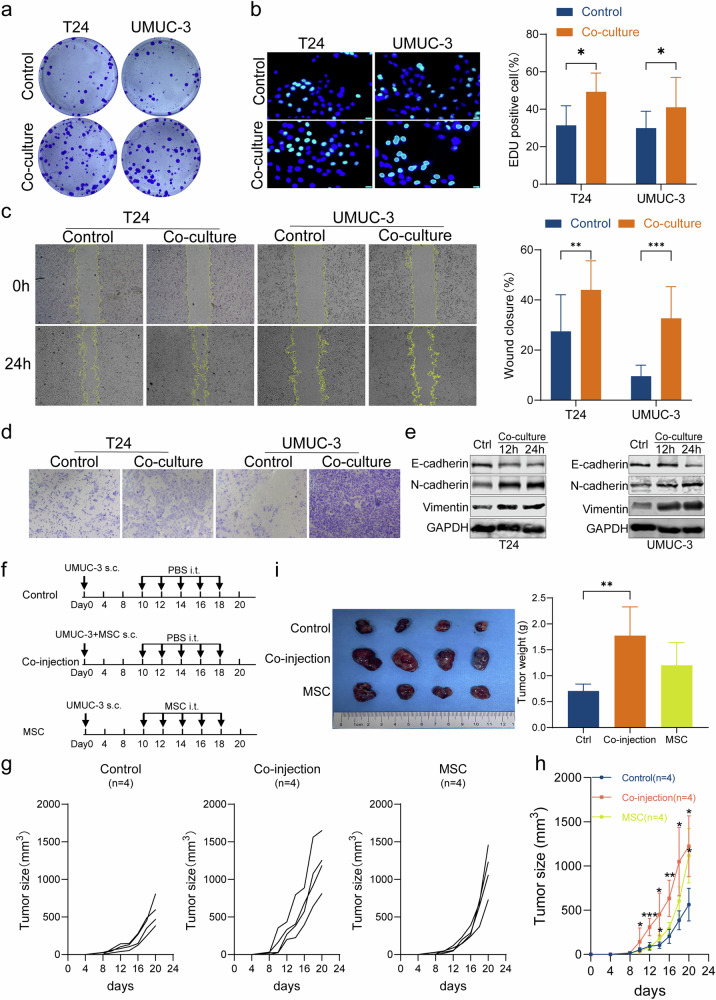
MSCs promote progression of bladder cancer cells in vitro and in vivo. The colony formation (**a**), EdU (**b**), wound healing (**c**), and transwell assays (**d**) showed a significant enhancement in bladder cancer cell proliferation, migration, and invasion abilities after co-culture with MSCs. Western blot analysis showed an increase in the expressions of N-cadherin and Vimentin, while a decrease in the expression of E-cadherin was observed after co-culture with MSCs (**e**). The animal treatment timeline(**f**). The tumor formation in the co-injection group was faster than the control (**g**). Tumors of the co-injection (*n* = 4) and MSCs group (*n* = 4) grew significantly more quickly than those in the control group (*n* = 4) at different time points (**h**). The net weight of tumors in the co-injection and MSC group was increased compared with that of the control (**i**). *P < 0.05, **P < 0.01, ***P < 0.001.

In order to evaluate the biological implications of MSC in BC progression in vitro, UMUC-3 and T24 cells were selected to co-culture with MSC in transwell co-culture plates. The results of the colony formation (Fig. 2a), EdU (Fig. 2b), wound healing (Fig. 2c), and transwell assays (Fig. 2d) demonstrated a significant enhancement in BC cell proliferation, migration, and invasion abilities after co-culture with MSCs (all *p* < 0.05). Subsequently, Western blot analysis was employed to investigate the expression patterns of epithelial-mesenchymal transition (EMT)-related factors (N-cadherin, E-cadherin, and Vimentin). The findings revealed an increase in the expressions of N-cadherin and Vimentin, while a decrease in the expression of E-cadherin was observed after co-culture with MSCs (Fig. 2e). These findings collectively demonstrated that MSCs increased the ability of BC cells to proliferate, migrate, and invade. Additionally, we sorted MSCs from BC patient tissues (p-MSCs) using a previously established gating strategy and assessed their differentiation potential (Fig. S1a). Under specific induction conditions, p-MSCs were able to differentiate into osteogenic (Fig. S1b), chondrogenic (Fig. S1c), and adipogenic (Fig. S1d) lineages. Colony formation assays showed that p-MSCs from two BC patients enhanced the proliferation of BC cells (Fig. S1e).

To evaluate the influence of MSC on tumorigenesis and tumor growth of human BC, a xenograft study of UMUC-3 cells was carried out using female athymic mice. The animal treatment timeline is illustrated in Fig. 2f. As shown in Fig. 2g, the tumor formation time in the co-injection group was shorter than control. Following tumor formation, tumors of the co-injection and MSCs group grew significantly more quickly than those in the control group at different time points (Fig. 2h). At the end of the experiment, the net weight of tumors in the co-injection and MSC group was increased compared with that of the control (Fig. 2i).

### MSCs enhance the quality and quantity of mitochondria to promote progression of bladder cancer

The biology of tumor cells is closely linked to their metabolic activities. Tumor cell metabolism is regulated by four main factors: the intrinsic metabolic activity of the tumor cells, interactions between tumor cells and other cells within the TME such as immune cells and stromal cells, the specific location and heterogeneity of the tumor, and the overall metabolic homeostasis of the host organism [11]. While previous studies have primarily focused on the impact of MSC-derived exosomes or cytokines on tumor cell signaling pathways [10], the effects of MSCs on tumor cell energy metabolism remain unclear. To clarify the effect of MSCs on cancer energy metabolism alteration, targeted detection of energy metabolomics of UMUC-3 cells with or without co-culturing with MSCs was performed. PCA revealed a distinct segregation of component distribution (Fig. S2a), indicating variations in energy metabolite composition between the two groups. In order to identify different metabolites between two groups, an S-plot was generated through implementation of an OPLS-DA model. Utilizing the OPLS-DA model, VIP was obtained and then 8 differential metabolites were identified (Fig. S2b and Table S2). Of note, the MSCs-treated group was characterized by a relative depletion of Krebs cycle substrates (BPG, Lactate, L-Alanine) and ATP synthesis substrates (ADP, AMP) when compared to control cells (Fig. S2c). These results suggest that MSCs may impact the mitochondrial energy metabolism of BC cells.

**Fig. 3:**
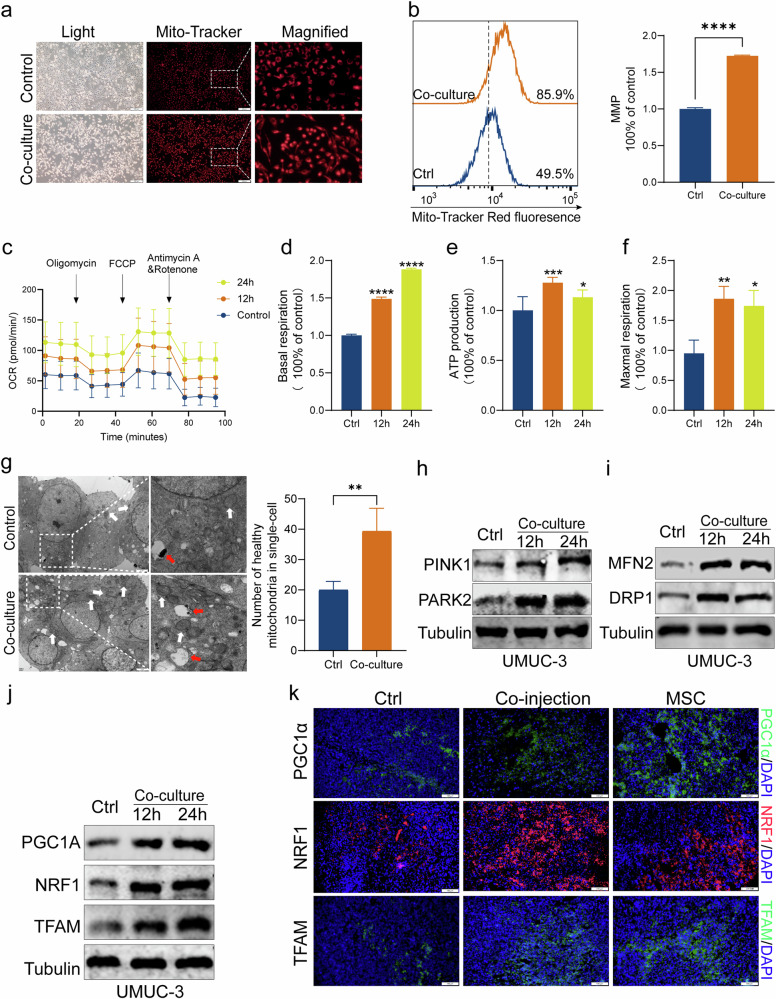
MSCs enhance the quality and quantity of mitochondria to promote progression of bladder cancer. Immunofluorescence and flow cytometry showed the MMP of UMUC-3 was augmented subsequent to co-culture with MSCs (**a**, **b**). Seahorse mito-stress assays showed the basal cellular OCR, ATP production, and maximum respiration were significantly elevated in UMUC3 cells after co-culture with MSCs compared to the control (**c**–**f**). Electron Microscopy of the UMUC-3 cancer cells showed an increase in the number of healthy mitochondria (white puncta) and in autophagic flux (red puncta) after co-cultured with MSCs (**g**). MSCs-treated cancer cells in vitro showed higher levels of PINK1, PARK2 (**h**), DRP1, TFAM (**i**), PGC1α, NRF1, and TFAM (**j**). Immunofluorescence of the tumor tissue showed the co-injection and MSC group had higher expression levels of PGC-1, NRF1, and TFAM than the control (**k**). *P < 0.05, **P < 0.01, ***P < 0.001, ****P < 0.0001.

To further validate whether the promoting effect of MSCs on BC energy metabolism alteration was achieved through the enhancement of mitochondrial metabolism, the MMP of UMUC-3 was estimated by immunofluorescence and flow cytometry following co-cultivation with MSCs. The results showed that the MMP of UMUC-3 was augmented subsequent to co-culture with MSCs (Fig. 3a, b). As shown in Fig. 3c–f, the basal cellular OCR, ATP production, and maximum respiration were significantly elevated in UMUC3 cells after co-culture with MSCs compared to the control. To determine whether increased OCR correlated with increased mitochondria number and mitochondrial mass, we measured mitochondrial density. Electron Microscopy of the UMUC-3 cancer cells showed an increase in the number of healthy mitochondria (white puncta) and in autophagic flux (red puncta) after co-cultured with MSCs (Fig. 3g). MSCs-treated cancer cells in vitro showed higher levels of mitophagy-linked proteins PINK1 and PARK2, which were responsible for eliminating unhealthy mitochondria to preserve optimal cellular damage control(Fig. 3h). The expression of DRP1, an essential protein in mitochondrial fission, and MFN2, a critical protein in mitochondrial fusion was also upregulated in MSCs-treated cancer cells (Fig. 3i). Further, the expression of PGC1α, a crucial regulator of mitochondrial biogenesis, and the downstream proteins NRF1 and TFAM were upregulated in MSCs-treated cancer cells (Fig. 3j). Similar results were found with immunofluorescence analysis of sections of tumor tissues from mice, which illustrated that the co-injection and MSC group had higher expression levels of the proteins associated with mitochondrial biogenesis (PGC-1α, NRF1, and TFAM) than the control (Fig. 3k). The above results indicate that MSCs enhance mitochondrial autophagy and mitochondrial biogenesis of BC cells to increase energy production, thereby promoting BC cell proliferation, migration, invasion, and EMT.

### MSCs activate AMPK pathway to enhance mitochondrial function of cancer cells

**Fig. 4:**
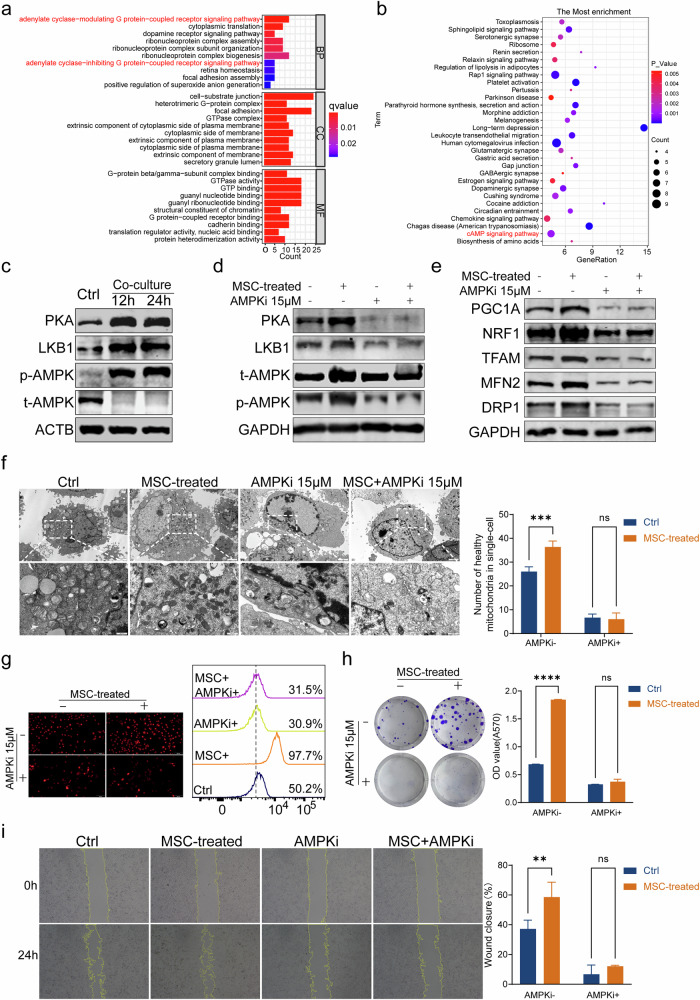
MSCs activate AMPK pathway to enhance mitochondrial function of cancer cells. GO (**a**) and KEGG pathway (**b**) enrichment analyses of the 73 differential expression proteins. Immunoblot showed that expression of PKA, LKB1 on UMUC-3 was significantly up-regulated and then phosphorylate AMPK after coculture with MSCs for 12 h and 24 h (**c**). AMPKi dorsomorphin effectively inhibited the AMPK pathway, thereby attenuating the activating effect of MSCs on AMPK (**d**). The promotion of MSCs to the expression of BC cells of PGC1α, NRF1, TFAM, DRP1, and MFN2 no longer exists in the presence of AMPKi (**e**). Improved mitochondrial morphology and increased mitochondrial numbers of BC caused by MSCs were disrupted by AMPKi (**f**). MMP hyperpolarization of UMUC-3 induced by MSCs could be disrupted by AMPKi (**g**). Colony formation assays (**h**) and wound closure assays(**i**) showed that AMPKi administration could decrease the proliferation and migration of BC and disrupt the pro-tumor effect of MSCs on BC. *P < 0.05, **P < 0.01, ***P < 0.001, ****P < 0.0001.

To further study the underlying mechanism of mitochondrial quality control, we performed the proteomic analysis to compare the proteomic differences between UMUC-3 co-cultured with or without MSCs. 3738 proteins were identified, 66 proteins were significantly upregulated, and 7 proteins were significantly downregulated in UMUC-3 cells treated with MSCs compared to control (|FC| > 2, *P* < 0.05). All differential expression proteins (DEPs) are summarized in Supplementary Table S3. A volcano plot and heat map were applied to show the DEPs selected with the above criterion (Fig. S3a, b). GO and KEGG pathway enrichment analyses of the 73 proteins were performed. Among the enriched biological processes, the protein−coupled receptor signaling pathway, coupled to cyclic nucleotide second messenger, adenylate cyclase modulating G protein-coupled receptor signaling pathway, and heterotrimeric G protein complex were at the top of the list (Fig. 4a). KEGG pathway analysis included enrichment in cAMP signaling pathway, chemokine signaling pathway, and Rap1 signaling pathway (Fig. 4b). G proteins are trimeric αβγ complexes, in which the α subunit Gαs can stimulate adenylyl cyclases enzymatic activity, resulting in an increase of intracellular cAMP levels [21]. PKA, the cAMP effector, can activate AMPK through LKB1 [21]. AMPK can regulate mitochondrial number through stimulation of mitochondrial biogenesis, regulation of the shape of the mitochondrial network in cells, and mitochondrial quality through regulation of autophagy and mitophagy [22]. Therefore, we hypothesized that MSCs activate AMPK via the PKA/LKB1 pathway to enhance the mitochondrial function of cancer cells.

Immunoblot analysis showed that the expression of PKA and LKB1 in UMUC-3 cells was significantly upregulated, leading to the phosphorylation of AMPK after co-culture with MSCs for 12 and 24 h (Fig. 4c). Next, we suppressed the pathway with AMPK inhibitor (AMPKi) dorsomorphin. The results indicate that AMPKi dorsomorphin effectively inhibited the AMPK pathway, thereby attenuating the activating effect of MSCs on AMPK (Fig. 4d). Additionally, immunoblot showed that the promotion of MSCs to the expression of BC cells of PGC1A, NRF1, TFAM, DRP1, and MFN2 no longer exists in the presence of AMPKi (Fig. 4e). Improved mitochondrial morphology and increased mitochondrial numbers of BC caused by MSCs were also disrupted by AMPKi (Fig. 4f). MMP hyperpolarization of UMUC-3 induced by MSCs could be disrupted by AMPKi (Fig. 4g). Colony formation assays and wound closure assays showed that AMPKi administration could decrease the proliferation and migration of BC and disrupt the pro-tumor effect of MSCs on BC cells (Fig. 4h–i). Similar results were obtained in T24 cells (Fig. S4). The above results demonstrated that MSCs could enhance mitochondrial function by activating the AMPK pathway thereby promoting the progression of BC.

### MSCs secrete kynurenine to activate AMPK pathway

**Fig. 5:**
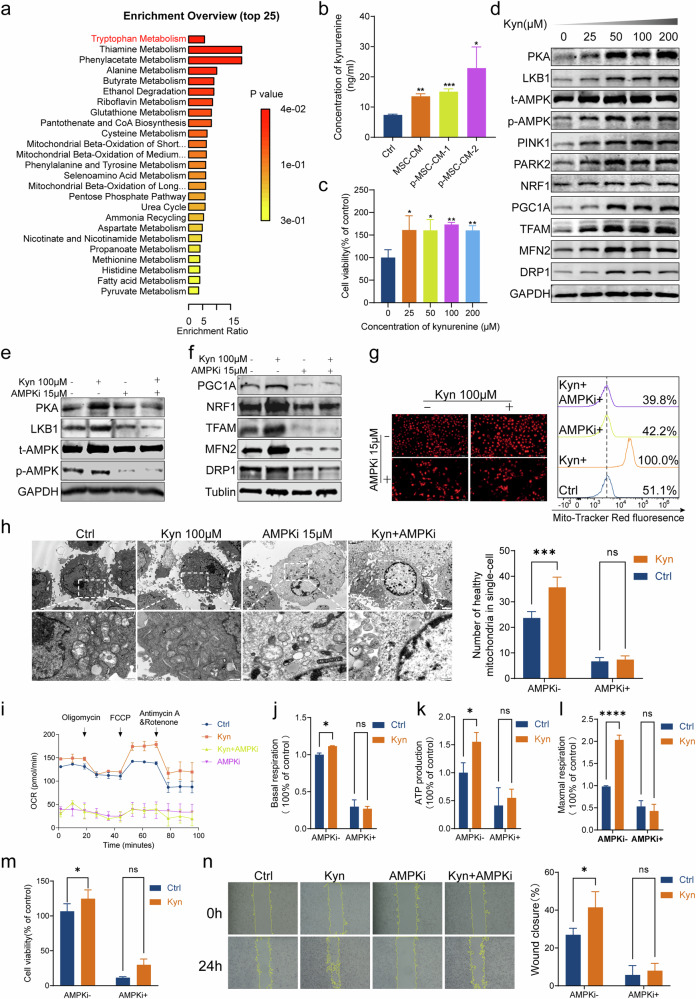
MSCs secrete kynurenine (Kyn) to activate AMPK pathway. Metabolic pathway and enrichment analysis using MetaboAnalyst(**a**). The concentrations of Kyn in MSC-CM were higher than those in the control medium (**b**). CCK8 assays and wound healing assays revealed that Kyn promoted UMUC-3 cell proliferation and migration (**c**, **n**). Immunoblot showed that the expression of PKA, LKB1, p-AMPK, PINK1, PARK2, PGC1α, NRF1, TFAM, DRP1, and MFN2 in UMUC-3 was increased after treatment with increasing concentrations of Kyn (**d**). AMPK inhibitor (AMPKi) dorsomorphin effectively inhibited the AMPK pathway, thereby attenuating the activating effect of Kyn on AMPK (**e**). Immunoblot showed that the expression-promoting effect of Kyn to PGC1α, NRF1, TFAM, DRP1, and MFN2 was prevented by the inhibition of AMPK (**f**). Kyn induced MMP hyperpolarization, enhanced mitochondrial morphology, and increase mitochondrial numbers, all of which were impeded by AMPKi (**g**, **h**). Mitostress assay demonstrated that Kyn significantly promoted mitochondrial metabolism (**i**), including a significant increase in basal cellular OCR (**j**), ATP production (**k**), and maximum respiration (**l**) in UMUC-3 cells compared to the control. CCK8 and wound closure assays showed that AMPKi could disrupt the pro-tumor effect of Kyn (**m**, **n**). *P < 0.05, **P < 0.01, ***P < 0.001, ****P < 0.0001.

To identify the molecules that promote tumorigenesis in MSC-CM, BC cell lines T24 and UMUC-3 were incubated with MSC-CM or a control medium at different percentages for 48 h. CCK8 assay showed that MSC-CM promoted the proliferation of cancer cells (Fig. S5a, b). Subsequently, MSC-CM was boiled to denature the proteins or subjected to ultracentrifugation to remove the exosome. Interestingly, both boiling CM and exosome-free CM were still able to promote the proliferation of cancer cells, although to a lesser extent than control MSC-CM (Fig. S5c). We thus hypothesized that the metabolites produced by MSC promoted the progression of BC. Non-targeted metabolomics analysis was conducted on the supernatants of MSCs to compare the different metabolites in MSC-CM and the control media. 24 upregulated differential metabolites were found between the two groups (Table S4). We next performed metabolic pathway and enrichment analysis using MetaboAnalyst. As seen in Fig. 5a, the most significantly affected pathways with lower *p* values and higher pathway impact were tryptophan metabolism, thiamine metabolism, and phenylacetate metabolism. According to a previous study [23], the introduction of exogenous kynurenine (Kyn) reinstated motility and clonogenic survival in the absence of tryptophan in glioma cells. Thus, we measured the concentrations of Kyn and found the concentrations of Kyn in MSC-CM and p-MSC-CM were higher than those in the control medium (Fig. 5b). Based on this observation, we assumed that MSCs secrete Kyn to promote the progression of BC cells.

To further validate the effects of Kyn on BC cells, UMUC-3 was treated with various concentrations of Kyn. CCK8 assays and wound healing assays revealed that Kyn promoted UMUC-3 cell proliferation and migration (Fig. 5c, n). We further explored whether the pro-tumor effects of Kyn were achieved by activating the AMPK pathway. Immunoblot showed that the expression of PKA, LKB1, and p-AMPK in UMUC-3 was increased after treatment with increasing concentrations of Kyn. Meanwhile, we observed that Kyn treatment led to elevated levels of PINK1, PARK2, PGC1A, NRF1, TFAM, DRP1, and MFN2 in vitro (Fig. 5d). In addition, AMPKi dorsomorphin effectively inhibited the AMPK pathway, thereby attenuating the activating effect of Kyn on AMPK (Fig. 5e). Immunoblot showed that the expression-promoting effect of Kyn to PGC1A, NRF1, TFAM, DRP1, and MFN2 was prevented by the inhibition of AMPK (Fig. 5f). Kyn was observed to induce MMP hyperpolarization, enhance mitochondrial morphology, and increase mitochondrial numbers, all of which were impeded by AMPKi (Fig. 5g, h). As shown in Fig. 5i–l, the mitostress assay demonstrated that Kyn significantly promoted mitochondrial metabolism (Fig. 5i), including a significant increase in basal cellular OCR(Fig. 5j), ATP production (Fig. 5k), and maximum respiration (Fig. 5l) in UMUC-3 cells compared to the control. This enhancement of mitochondrial metabolism by Kyn was reversed by the AMPKi. CCK8 and wound closure assays showed that AMPKi could disrupt the pro-tumor effect of Kyn (Fig. 5m, n). In T24 cells, we similarly found that Kyn promotes mitochondrial homeostasis through the AMPK pathway to promote BC progression (Fig. S6). The above results demonstrated that MSCs produced Kyn, which could enhance mitochondrial function by activating the AMPK pathway thereby promoting the progression of BC.

### Kyn level in urine positively correlated with the malignancy of BC

**Fig. 6:**
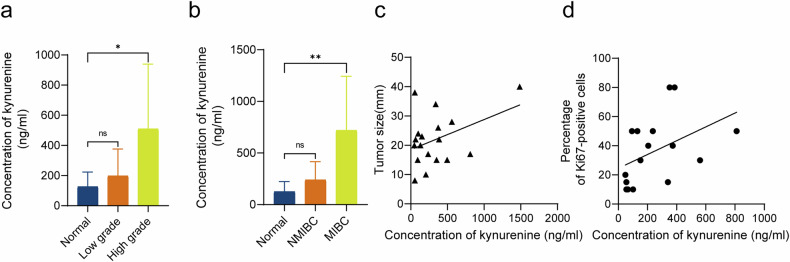
Kyn level in urine positively correlated with the malignancy of bladder cancer. The concentration of Kyn in the high-grade group was significantly higher than that in the normal group (**a**). The concentration of Kyn in the MIBC group was significantly higher than that in the normal group (**b**). The Kyn levels in urine were positively correlated with tumor size (**c**) and the percentage of Ki67-positive cells (**d**). *P < 0.05, **P < 0.01.

To determine whether Kyn was clinically relevant in BC, we examined concentrations of Kyn in 26 urine samples including 18 BC patients and 8 healthy volunteers. As shown in Fig. 6a, compared with the normal group, the concentration of Kyn in the high-grade group was significantly higher (*P* < 0.05), while no significant changes in low-grade group were observed. Similarly, compared with the normal group, the concentration of Kyn in the muscle-invasive bladder cancer(MIBC) group was significantly higher (*P* < 0.05), while no significant changes in the non-muscle-invasive bladder cancer(NMIBC) group were observed (Fig. 6b). Using Pearson’s correlation test, we further analyzed the correlation between concentrations of Kyn in urine and tumor size or percentage of Ki67-positive cells. The Kyn levels in urine were positively correlated with tumor size (Fig. 6c, *r* = 0.41) and the percentage of Ki67-positive cells (Fig. 6d, *r* = 0.44). These results indicate that the Kyn levels in urine are positively correlated with the malignancy of BC, which might contribute to BC progression.

### IDO1 inhibitor could reverse the tumor‑promoting effects of MSCs in vitro and vivo

**Fig. 7:**
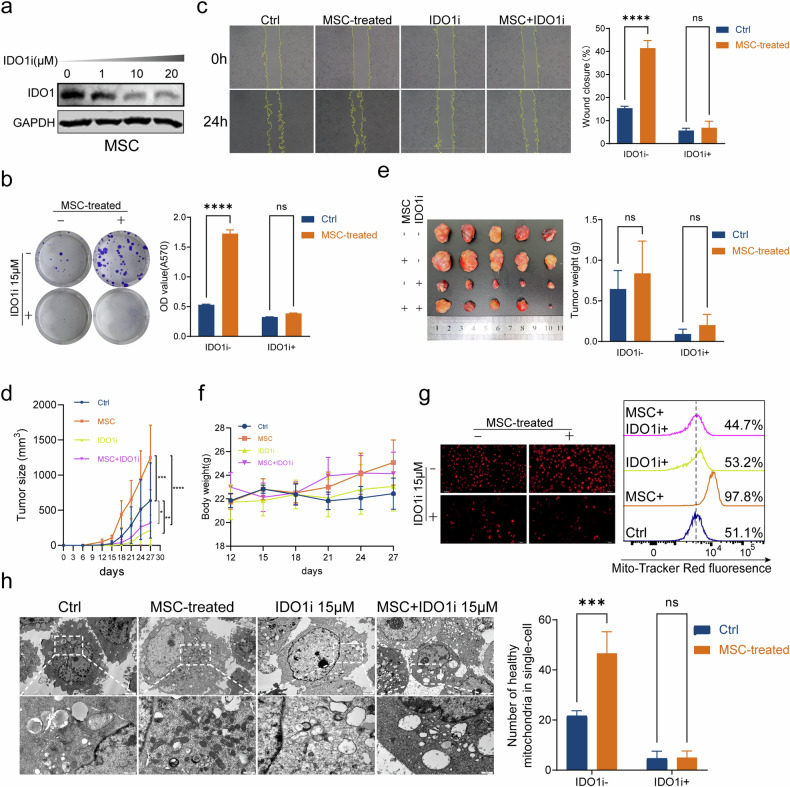
IDO1 inhibitor (IDO1i) could reverse the tumor‑promoting effects of MSCs in vitro and in vivo. Immunoblot showed that the expression of IDO1 decreased significantly in MSCs with the increased concentration of linrodostat (**a**). Colony formation assays (**b**) and wound-healing assays (**c**) confirmed that IDO1i can impede the pro-tumorigenic effects of MSCs in UMUC-3 cells. The growth rate of tumor volume was slow in IDO1i and MSC+IDO1i groups (**d**), and tumor weight also showed a significant decrease in IDO1i and MSC+IDO1i groups (**e**). No significant difference in body weight was observed in four groups (**f**). In the presence of linrodostat, MSCs failed to induce MMP hyperpolarization in UMUC-3 cells (**g**). TEM showed that MSCs improved mitochondrial morphology and increased mitochondrial numbers, which could be disrupted by linrodostat (**h**). *P < 0.05, **P < 0.01, ***P < 0.001, ****P < 0.0001.

IDO1 (indoleamine 2,3-dioxygenase 1) was an enzyme that played a crucial role in the metabolism of the essential amino acid tryptophan [24]. We next thought if an IDO1 inhibitor could use to downregulate IDO1 in MSCs to decrease Kyn production and reverse the pro-tumorigenic effects of Kyn secretion from MSCs on BC. MSCs were treated with different concentrations of IDO1 inhibitor (IDO1i) linrodostat for 24 h, and it was observed that the expression of IDO1 decreased significantly in MSCs with the increased concentration of IDO1i (Fig. 7a). Next, we evaluated whether the IDO1 inhibitor could reverse the pro-tumorigenic effects of MSCs. Colony formation assays and wound-healing assays confirmed that the pro-tumorigenic effects of MSCs can be impeded in UMUC-3 cells (Fig. 7b, c). To verify the above results in vivo, a nude mouse xenograft experiment was performed. Linrodostat or PBS was injected intra-peritoneal into mice 5 times every 3 days. Interestingly, the growth rate of tumor volume was slow in IDO1i and MSC+IDO1i groups (Fig. 7d), and tumor weight also showed a significant decrease in IDO1i and MSC+IDO1i groups (Fig. 7e). To assess the safety of linrodostat, mice’s body weight was monitored and no significant difference was observed in four groups (Fig. 7f). We further explored whether the reversal effects of IDO1i on the pro-tumor activities of MSCs were achieved by regulating the quality and quantity of mitochondria. In the presence of IDO1i, MSCs failed to induce MMP hyperpolarization in UMUC-3 cells (Fig. 7g). Furthermore, TEM confirmed that MSCs improved mitochondrial morphology and increased mitochondrial numbers, which could be disrupted by IDO1i (Fig. 7h). In T24 cells, IDO1 inhibition similarly reversed the promotion of BC by MSCs, with this effect associated with mitochondrial regulation (Fig. S7). To confirm that the effects of the IDO1i on cancer cell proliferation and migration are indeed mediated through the inhibition of Kyn production by MSCs, we used siRNA to knock down IDO1 expression in MSCs, which was confirmed by Western blot analysis (Fig. S8a). We then co-cultured si-IDO1 MSCs with BC cells. Compared to the control, the tumor-promoting ability of si-IDO1 MSCs was significantly reduced, with si-IDO1#2 showing the most pronounced effect (Fig. S8b). However, when Kyn was added back, the proliferation and migration abilities of the cancer cells were restored (Fig. S8c, d).

To verify the overall safety of IDO1i linrodostat in vivo, HE staining was used to observe the morphology of heart, liver, spleen, lung, kidney, intestine, and testes tissues. Staining showed no significant drug-related damage to main organs (Fig. S9).

## Discussion

TME exerts a pivotal role in driving tumor progression and modulating the response to cancer therapies [25]. Consequently, the development of therapeutic strategies targeting the intricate components and interactions within the TME holds significance in the field of oncology [25]. In the present study, we demonstrated that MSCs promote tumor proliferation and migration through the augmentation of energy production in BC cells. This enhancement is achieved by upregulating mitochondrial biogenesis via the activation of the PKA/LKB/AMPK/PGC1α axis. The process of mitochondrial biogenesis is intricate and encompasses the synthesis of both the inner and outer mitochondrial membranes, the synthesis of proteins encoded by the mitochondria, the import of proteins encoded by the nucleus into the mitochondria, and the replication of mitochondrial DNA (mtDNA) [26]. This process requires the coordinated regulation of two separate genomes, the nuclear genome and the mitochondrial genome. PGC-1α serves as a central regulator of mitochondrial biogenesis, orchestrating the transcriptional machinery to enhance mitochondrial number and enable cell adaptation to heightened energetic demands [26]. AMPK plays a significant role in PGC-1α phosphorylation [27]. In addition, AMPK can elevate NAD levels, leading to the phosphorylation of SIRT1. Once activated, SIRT1 deacetylates PGC-1α, thereby facilitating mitochondrial biogenesis [28]. The active form of PGC-1α translocates into the nucleus, where it activates NRF1 and NRF2 and then leads to the transcription of TFAM, thereby promoting the synthesis of mitochondrial proteins, replication and transcription of mtDNA, and the generation of new mitochondria [26, 29]. The findings of our study indicate that Kyn released by MSCs activates the AMPK pathway, thereby facilitating the expression of PGC1α, NRF1, and TFAM, which in turn promote mitochondrial biogenesis. Despite exhibiting heightened glycolysis, cancer cells also employ mitochondrial respiration as a means to obtain a substantial portion of their ATP [30]. Mitochondrial biogenesis caused by MSCs is helpful for meeting the energetic demands of cancer cells.

Furthermore, we found that MSCs maintained mitochondrial homeostasis by influencing mitochondrial fission, fusion, and mitophagy. Under conditions of mitochondrial damage and depolarization, DRP1 is recruited to the site of fission and begins to bisect the mitochondria into two pieces thereby isolating damaged mitochondria away from the healthy pool [31]. Mitochondrial fusion is ensured by MFN1 and MFN2, which mediate outer mitochondrial membrane fusion [31]. It is also known that mitochondrial dynamins are closely associated with increases in mitophagy [32]. Mitophagy regulators such as PINK1 and PARK2 are responsible for eliminating unhealthy mitochondria to preserve optimal cellular damage control. In this study, we observed that MSCs or Kyn treatment led to increasing levels of DRP1, MFN2, PINK1, and PARK2.

We chose adipose-derived hTERT-MSCs for our investigation based on the following considerations: Firstly, a meta-analysis has demonstrated that compared to patients with a normal body mass index (BMI), obese individuals have a higher risk of disease progression and recurrence [33]. Dose-response analysis revealed a linear correlation between BMI and recurrence risk, with each 1 kg/m² increase in BMI associated with a 1.3% increase in the risk of BC recurrence [34]. Secondly, during radical cystectomy, it was observed that patients often have a thick perivesical fat layer. The cells within this layer may have an unclear impact on the progression of BC. Thirdly, Kyn, as a functional substance promoting BC progression, is significantly elevated in the supernatant of MSCs derived from both BC tissue and adipose tissue (Fig. 5b). Given this observation, we chose to use the more stable adipose-derived hTERT-MSCs for mechanistic studies. The hTERT-MSCs exhibit all the characteristics and attributes usually associated with primary MSCs. However, hTERT immortalization also provides the benefit of longevity and expansion capabilities, eliminating concerns with donor variability.

There is mounting evidence indicating that MSCs directly support tumor cells through exosomes or cytokines [10]. From a metabolic perspective, we demonstrate that the tryptophan metabolite Kyn from MSCs promotes the progression of BC. In addition, we found that Kyn levels in urine are positively correlated with the malignancy of BC. Consistent with our results, other studies have shown that both plasma and urinary Kyn-Trp ratios are significantly elevated in BC patients [35]. Metabolic intercommunication between stromal cells and tumor cells is a crucial factor in the initiation, proliferation, and progression of cancer [11]. For example, CAFs have been shown to impact cancer metabolism by locally modulating metabolites such as lactate, glutamine, and aspartate [11]. The results of our study offer new insight into the mechanism through which MSCs contribute to tumor promotion.

The close association between cancer risks and a wider range of metabolites in biofluids, including blood, serum, urine, saliva, and sweat, has been established [36]. Urine metabolites, in particular, have been explored as potential cancer biomarkers, with significantly lower levels of citrate, 2,5-furandicarboxylic acid, ribitol, and ribonic acid observed in the urine of BC patients compared to healthy individuals [37–39]. Conversely, two independent studies have reported elevated levels of taurine in the urine of BC patients [39, 40]. Our study demonstrated that patients with BC, specifically those with MIBC or high-grade BC, exhibit a significantly increased concentration of Kyn in their urine samples when compared to healthy individuals. Furthermore, the concentration of Kyn in the urine samples of BC patients was found to be positively correlated with larger tumor volume and higher T stage, pathological grade, and percentage of Ki67-positive cells. These data provide evidence from clinical samples that Kyn promotes the progression of cancer. Kyn promotes tumor-cell survival and motility through the AHR in an autocrine/paracrine fashion in glioma [23]. The interaction between Kyn and AHR influences the formation of regulatory T cells [41]. Kyn activates AHR to promote the initiation of tumorigenesis but also its promotion, progression, and metastasis [41, 42]. We found another mechanism that Kyn could the quality and quantity of mitochondria by activating the AMPK pathway thereby promoting the progression of BC.

Based on our findings, we proposed a management strategy to reverse the pro-tumor effect of MSCs on BC and verified the efficiency and safety by further experiments in vivo and in vitro. The overexpression of IDO1 in both tumor cells and immune cells within the TME is a common occurrence in cancer [43]. This overexpression results in heightened tryptophan catabolism and the generation of immunosuppressive metabolites, including Kyn, which can impede the function of immune cells such as T cells and natural killer cells, thus facilitating immune evasion by cancer cells [24]. The targeting of IDO1 can augment the anti-tumor immune response and enhance the effectiveness of cancer immunotherapy [24, 44]. In the context of metastatic urothelial carcinoma, the combination of linrodostat and nivolumab in PD-1/PD-L1 inhibitor-naive patients who had undergone one or more previous therapies showed a promising overall response rate of 37% (with three complete responses and seven partial responses out of 27 patients) and a disease control rate of 56% (NCT03661320) [45]. Additionally, humanized MSCs expressing IDO were able to repress T lymphocyte proliferation [46]. In this study, we propose that an IDO1-targeted inhibitor can inhibit the production of Kyn, a product of MSCs’ tryptophan metabolism, in the TME, thereby reversing the pro-tumor effects of MSCs on BC. Combining the findings of the previous and present studies, we believe that IDO1 inhibitors can inhibit tumor progression through multiple mechanisms, including direct targeting of tumor cell IDO1, inhibition of the anti-tumor immune response, and improvement of the efficacy of cancer immunotherapy, as well as reversing the pro-tumor effects of MSCs.

## Conclusion

In summary, MSCs in TME produce tryptophan metabolites Kyn to enhance mitochondrial function by activating the AMPK pathway, thereby promoting BC progression. IDO1 inhibitor could effectively reverse the tumor‑promoting effects of MSCs on BC.

## Supplementary information

Supplemental Figures

Supplemental Table1

Supplemental Table2

Supplemental Table3

Supplemental Table4

Supplementary table legends

Supplementary material-Original image

## Data Availability

The datasets used and/or analysed during the current study are available from the corresponding author on reasonable request.
