# Supplementary material for: Mesenchymal stem cells in tumor microenvironment: drivers of bladder cancer progression through mitochondrial dynamics and energy production
Source: Cell Death Dis. 2024 Sep 20;15(9):688. doi: 10.1038/s41419-024-07068-9 (PMC11415494; doi:10.1038/s41419-024-07068-9)
Supplement: Supplementary file 1 — Supplemental Figures [file 41419_2024_7068_MOESM1_ESM.pdf]

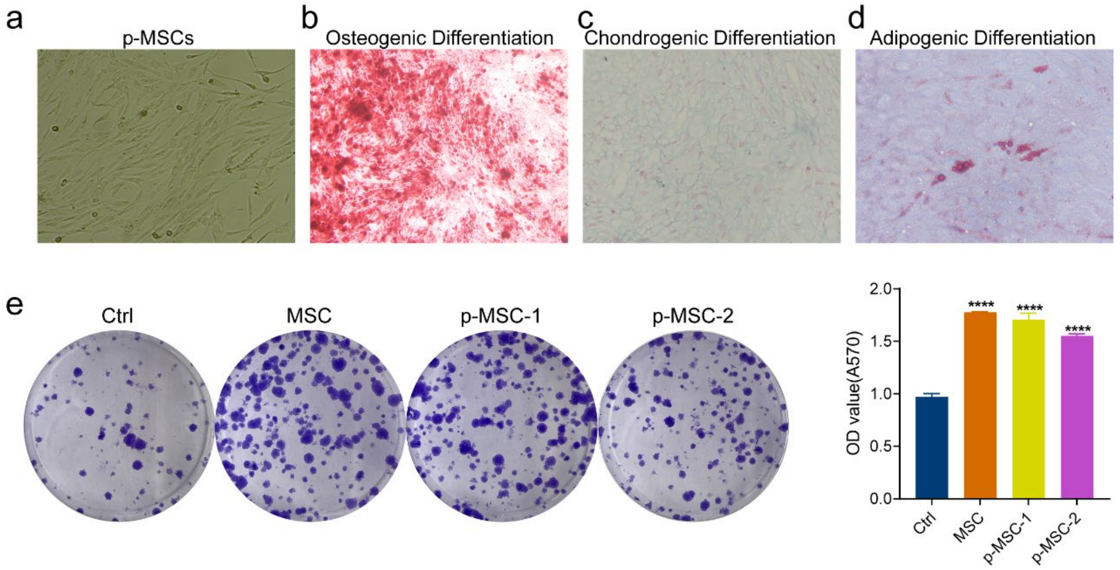

Supplementary Figure 1. MSCs derived from human bladder cancer tissues promote bladder cancer proliferation. MSCs from bladder cancer patient tissues (p-MSCs) were sorted by flow cytometry (a). These p-MSCs can induce differentiation into osteogenic(b), chondrogenic(c), and adipogenic (d) lineages. Colony formation assays show that p-MSCs from two bladder cancer patients enhance the proliferation of bladder cancer cells(e). $P < 0.001$ (***), $P < 0.0001$ (****).

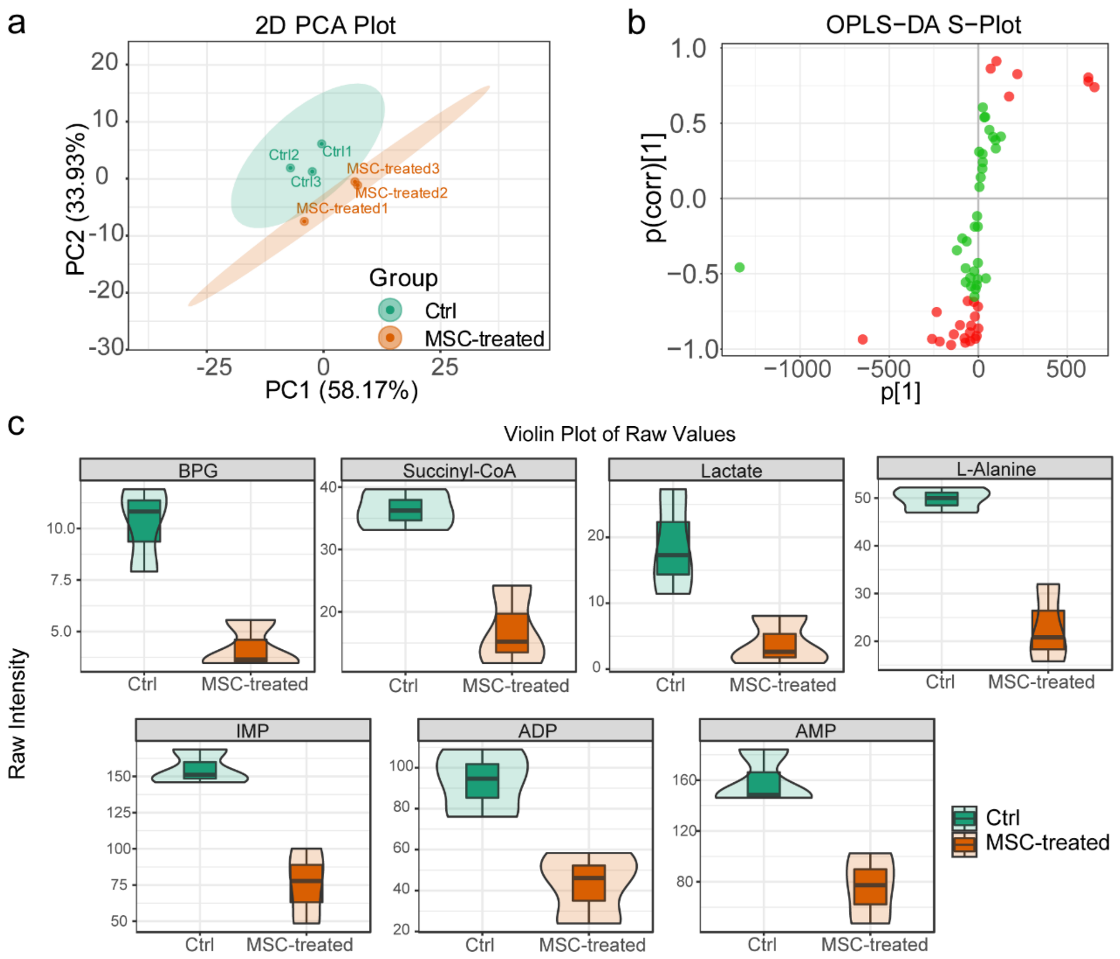

Supplementary Figure 2. Targeted detection of energy metabolomics showed that MSCs may impact the mitochondrial energy metabolism of BC cells. Principal component analysis (PCA) revealed a distinct segregation of component distribution (a). Utilizing the implementation of an orthogonal partial least-squares discriminant analysis (OPLS-DA) model, 8 differential metabolites were identified (b).

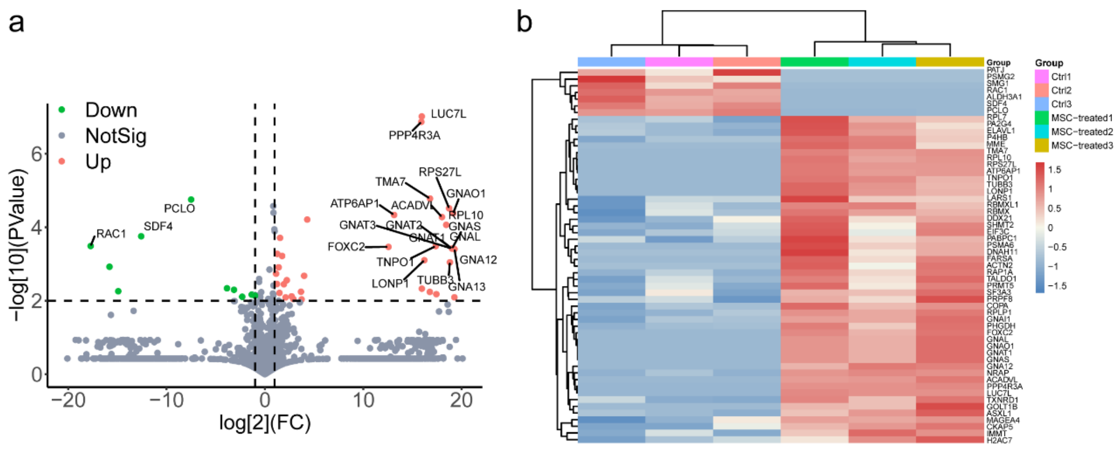

Supplementary Figure 3. A volcano plot(a) and heat map(b) showed the differential expression proteins.

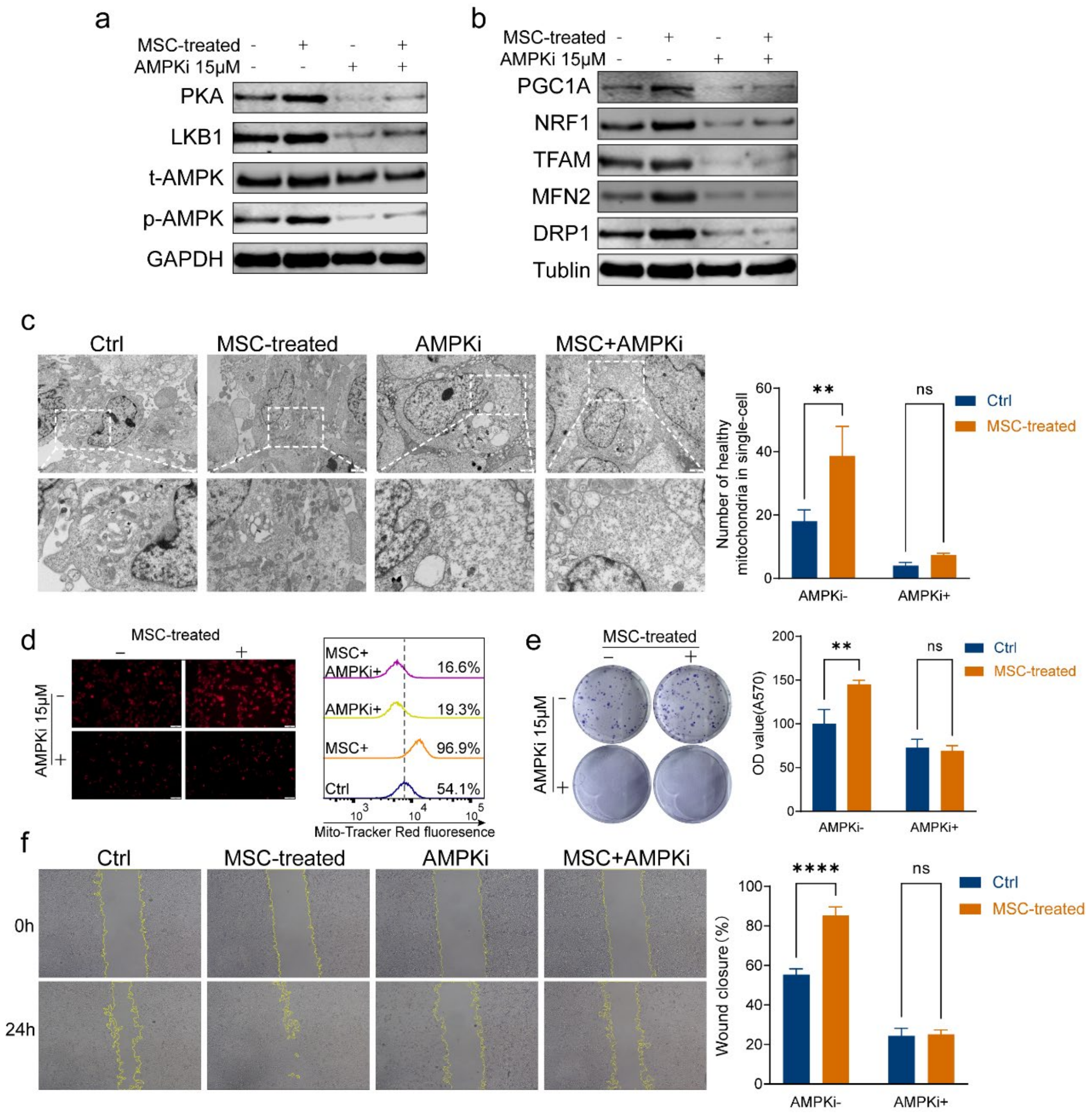

Supplementary Figure 4. MSCs activate AMPK pathway to enhance mitochondrial function in T24 cells. Immunoblot showed that expression of PKA, LKB1 in T24 was significantly up-regulated and then phosphorylate AMPK after coculture with MSCs. AMPKi dorsomorphin effectively inhibited the AMPK pathway, thereby attenuating the activating effect of MSCs on AMPK (a). The promotion of MSCs to the expression of T24 cells of PGC1A, NRF1, TFAM, DRP1, and MFN2 no longer exists in the presence of AMPKi (b). Improved mitochondrial morphology and increased mitochondrial numbers of BC caused by MSCs were disrupted by AMPKi (c). MMP hyperpolarization of T24 induced by MSCs could be disrupted by AMPKi (d). Colony formation assays(e) and wound closure assays(f) showed that AMPKi administration could decrease the proliferation and migration of BC and disrupt the pro-tumor effect of MSCs on BC. $P < 0.05$ (*), $P < 0.01$ (**), $P < 0.001$ (***), $P < 0.0001$ (****).

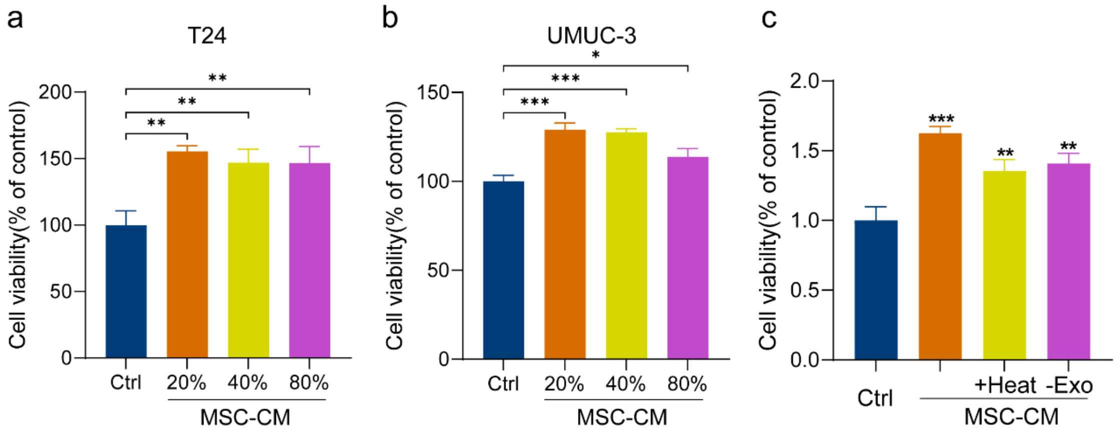

Supplementary Figure 5. CCK8 assay showed that MSC-CM promoted the proliferation of cancer cells (a-b). Both boiling CM and exosome-free CM were still able to promote the proliferation of cancer cells (c).

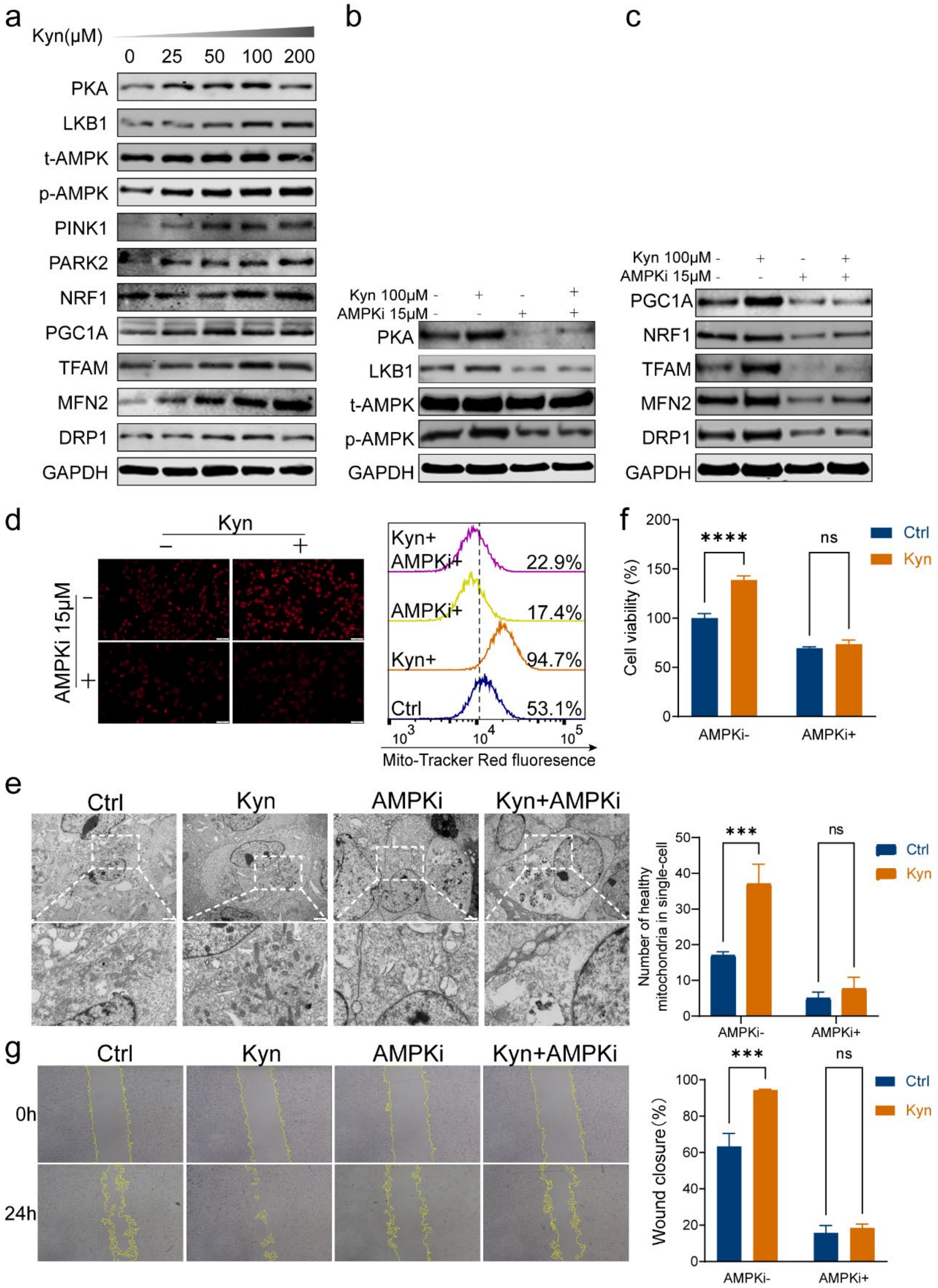

Supplementary Figure 6. MSCs secrete kynurenine (Kyn) to activate AMPK pathway in T24 cells. Immunoblot showed that the expression of PKA, LKB1, p-AMPK, PINK1, PARK2, PGC1A, NRF1, TFAM, DRP1, and MFN2 in UMUC-3 was increased after treatment with increasing concentrations of Kyn(a). AMPK inhibitor (AMPKi) dorsomorphin effectively inhibited the AMPK pathway, thereby attenuating the activating effect of Kyn on AMPK (b). Immunoblot showed that the expression-promoting effect of Kyn to PGC1A, NRF1, TFAM, DRP1, and MFN2 was prevented by the inhibition of AMPK

(c). Kyn induced MMP hyperpolarization, enhanced mitochondrial morphology, and increase mitochondrial numbers, all of which were impeded by AMPKi (d, e). CCK8 (f) and wound closure assays(g) showed that AMPKi could disrupt the pro-tumor effect of Kyn. $P < 0.05$ (*), $P < 0.01$ (**), $P < 0.001$ (***), $P < 0.0001$ (****).

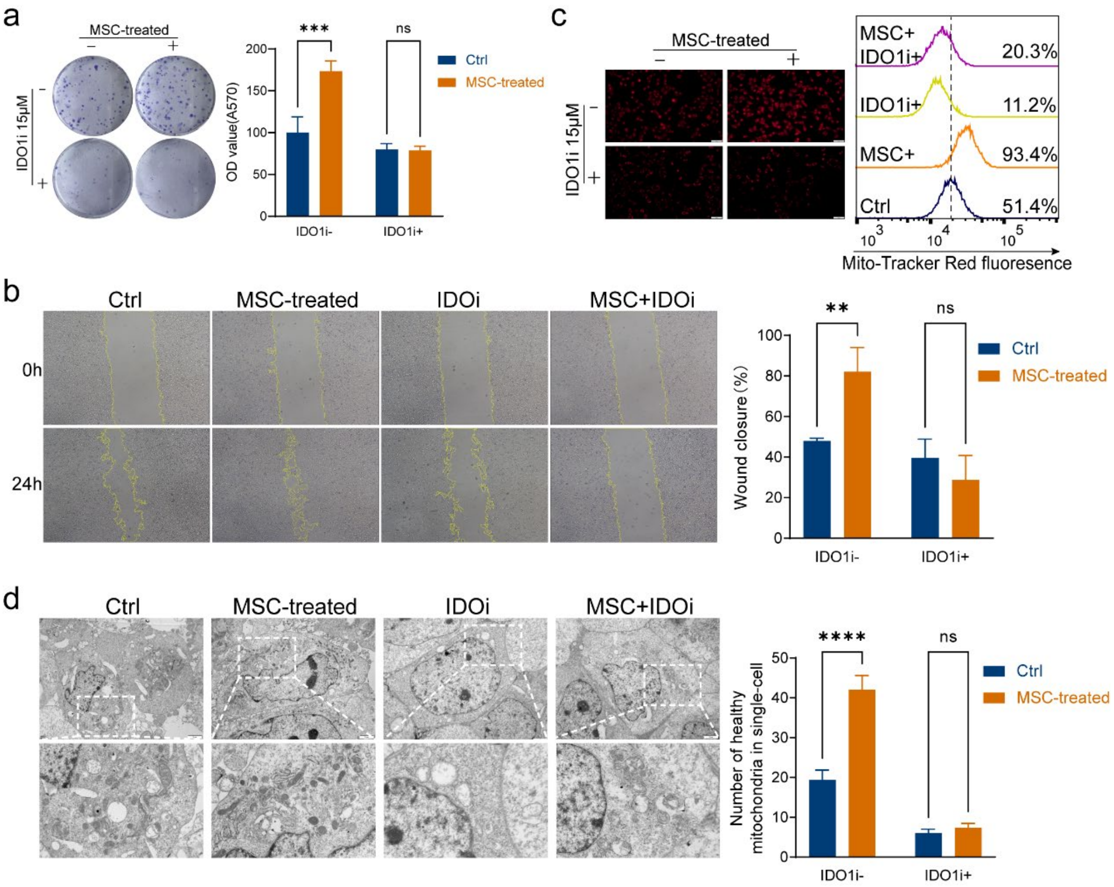

Supplementary Figure 7. IDO1 inhibitor (IDO1i) could reverse the tumor-promoting effects of MSCs in T24 cells. Colony formation assays (a) and wound-healing assays (b) confirmed that the pro-tumorigenic effects of MSCs can be impeded in T24 cells. In the presence of linrodostat, MSCs failed to induce MMP hyperpolarization in T24 cells (c). TEM showed that MSCs improved mitochondrial morphology and increased mitochondrial numbers, which could be disrupted by linrodostat (d). $P < 0.05$ (*), $P < 0.01$ (**), $P < 0.001$ (***), $P < 0.0001$ (****).

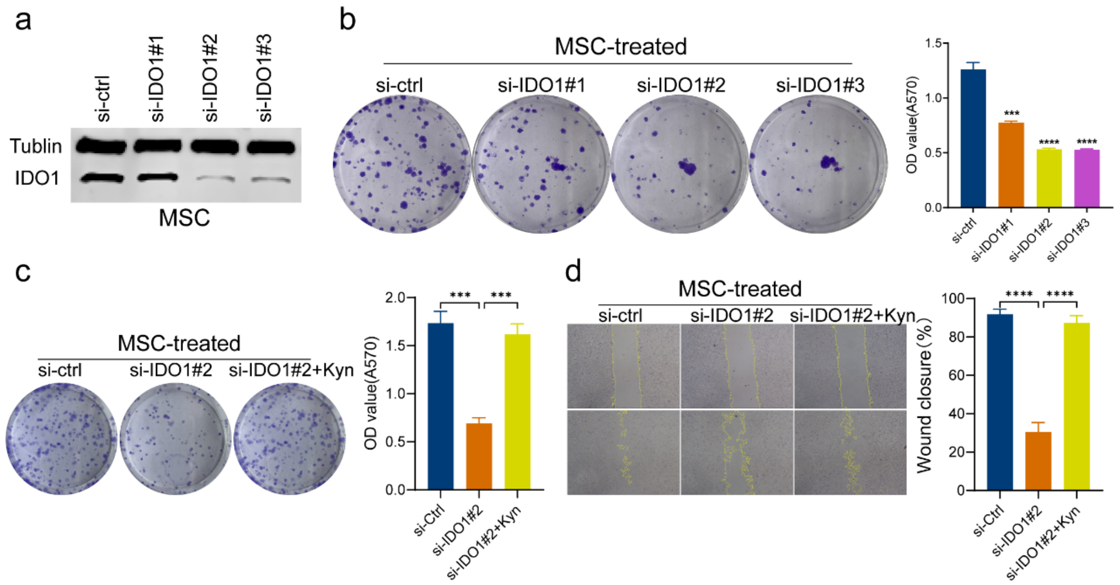

Supplementary Figure 8. Confirmation of IDO1 knockdown in MSCs by Western blot (a). IDO1-knockdown MSCs significantly reduced cancer cell proliferation compared to control MSCs (b). The addition of Kyn to the co-culture restored the proliferative capacity of the cancer cells (c). IDO1-knockdown MSCs without Kyn led to a significant reduction in cancer cell migration. The addition of Kyn reversed this effect (d). $P < 0.001$ (***), $P < 0.0001$ (****).

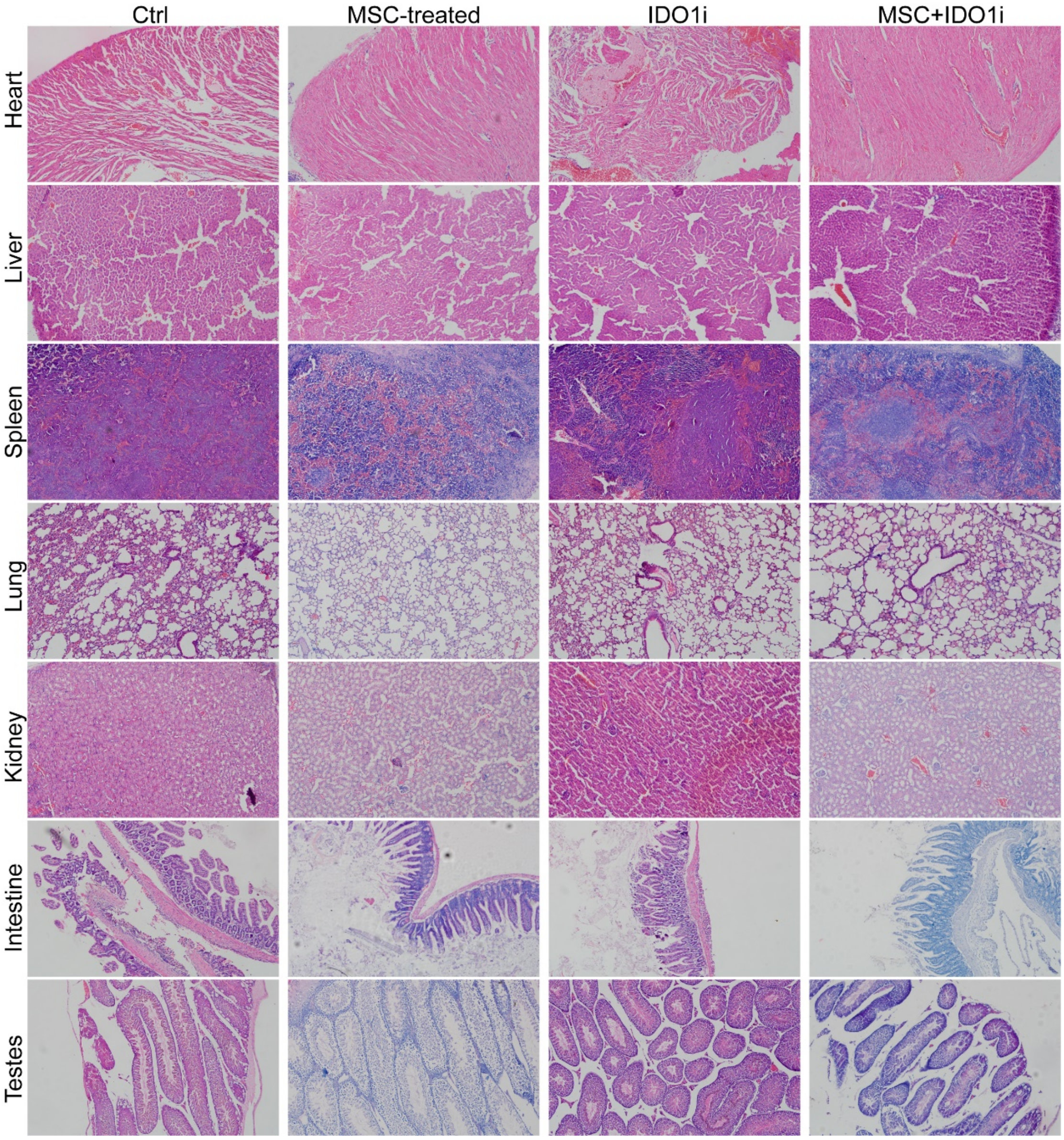

Supplementary Figure S9. H&E staining of mice heart, liver, spleen, lung, kidney, intestine, and testes tissues.
