# Supplementary material for: Mesenchymal stem cells in tumor microenvironment: drivers of bladder cancer progression through mitochondrial dynamics and energy production
Source: Cell Death Dis. 2024 Sep 20;15(9):688. doi: 10.1038/s41419-024-07068-9 (PMC11415494; doi:10.1038/s41419-024-07068-9)
Supplement: Supplementary file 6 — Supplementary table legends [file 41419_2024_7068_MOESM6_ESM.docx]

# Supplementary table legends

**Supplementary Table1:** All siRNA oligonucleotide sequences.

**Supplementary Table2:** The list of differential metabolites identified by targeted detection of energy metabolomics.

**Supplementary Table3:** The list of differential expression proteins identified by proteomic analysis.

**Supplementary Table4:** The list of 24 up-regulated differential metabolites between the MSC-CM and control medium.
