# Supplementary material for: Mesenchymal stem cells in tumor microenvironment: drivers of bladder cancer progression through mitochondrial dynamics and energy production
Source: Cell Death Dis. 2024 Sep 20;15(9):688. doi: 10.1038/s41419-024-07068-9 (PMC11415494; doi:10.1038/s41419-024-07068-9)

Figure 2e

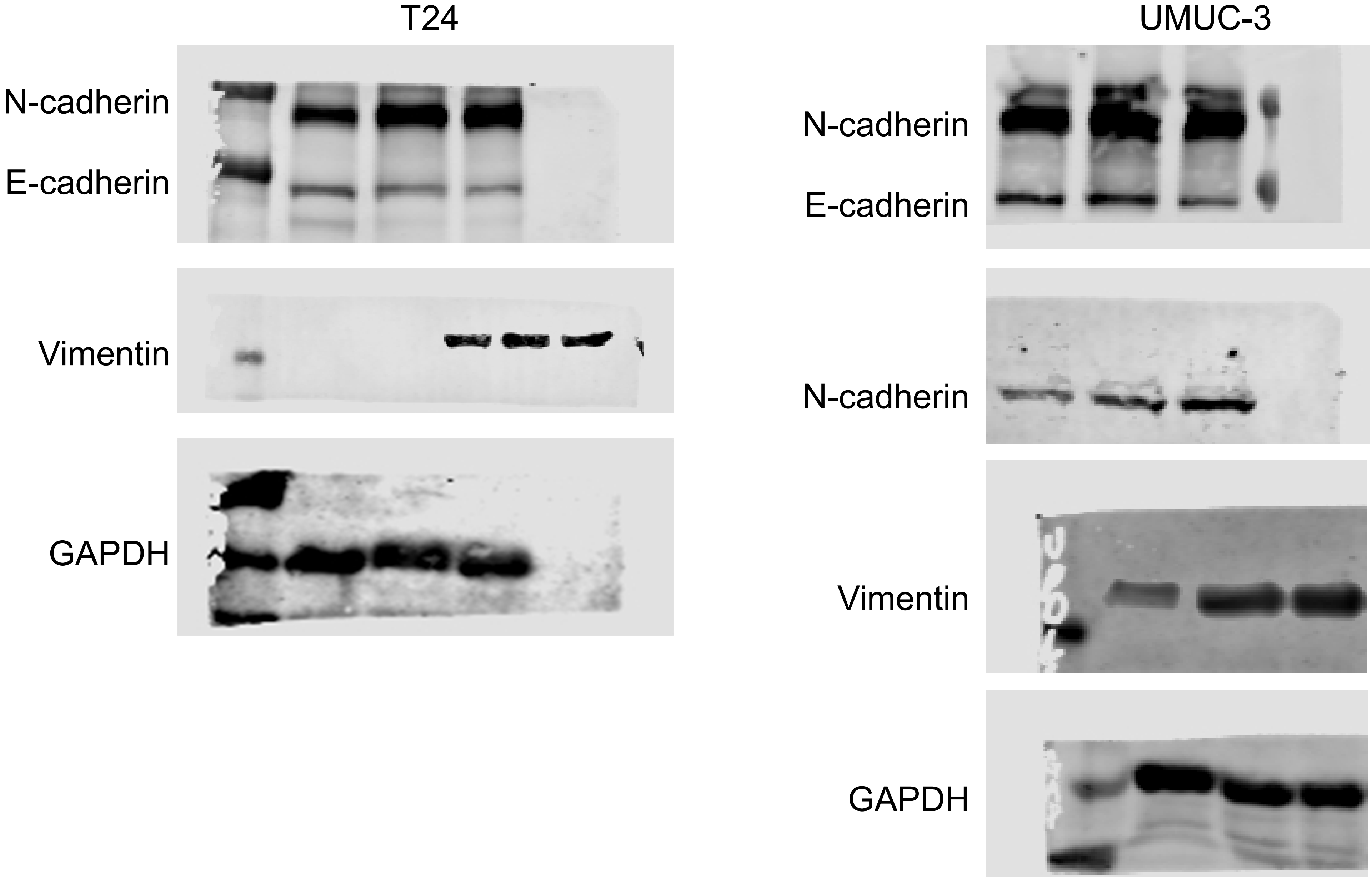
T24
N-cadherin
E-cadherin
Vimentin
GAPDH
UMUC-3
N-cadherin
E-cadherin
N-cadherin
Vimentin
GAPDH

Figure 4h

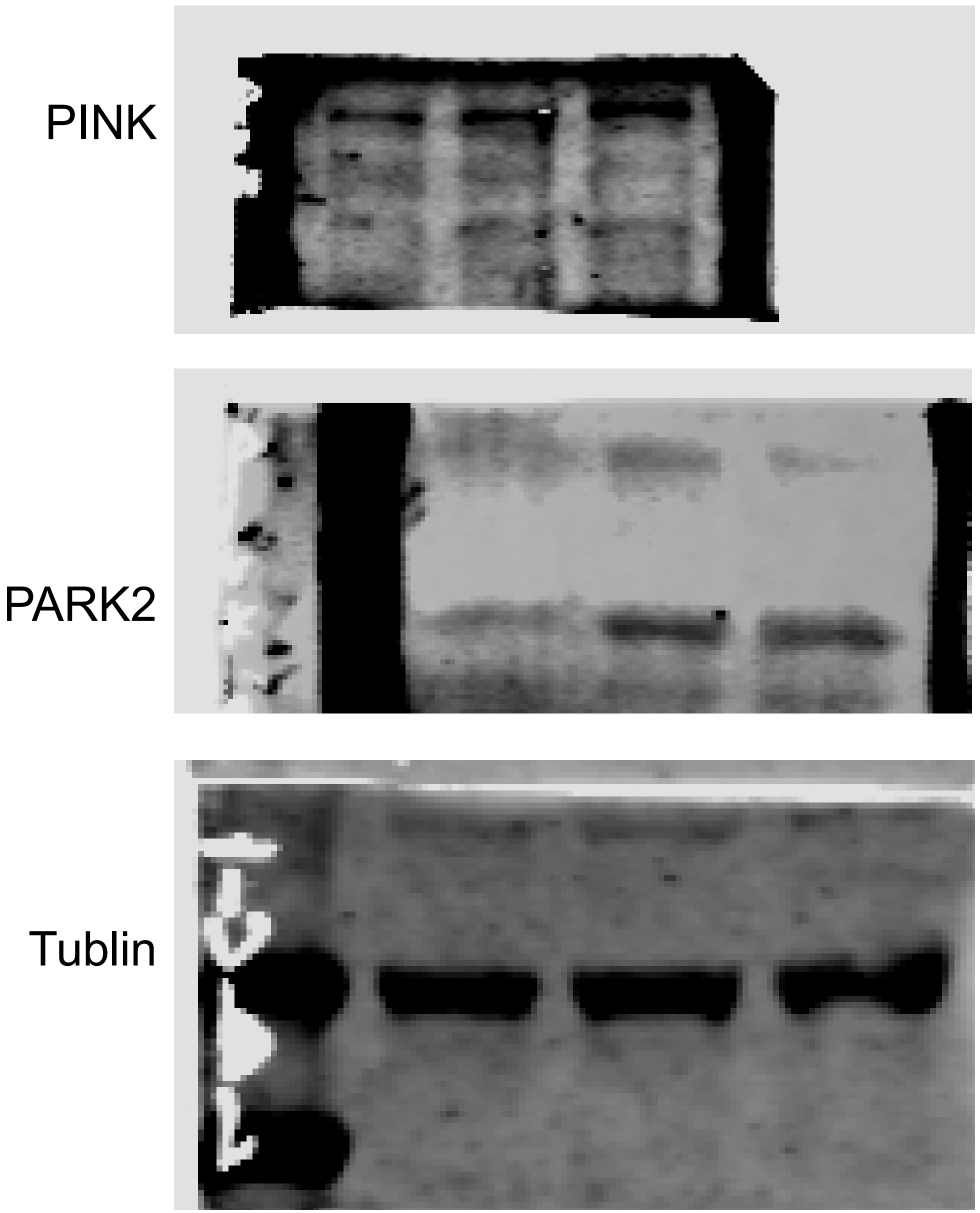

Figure 4i

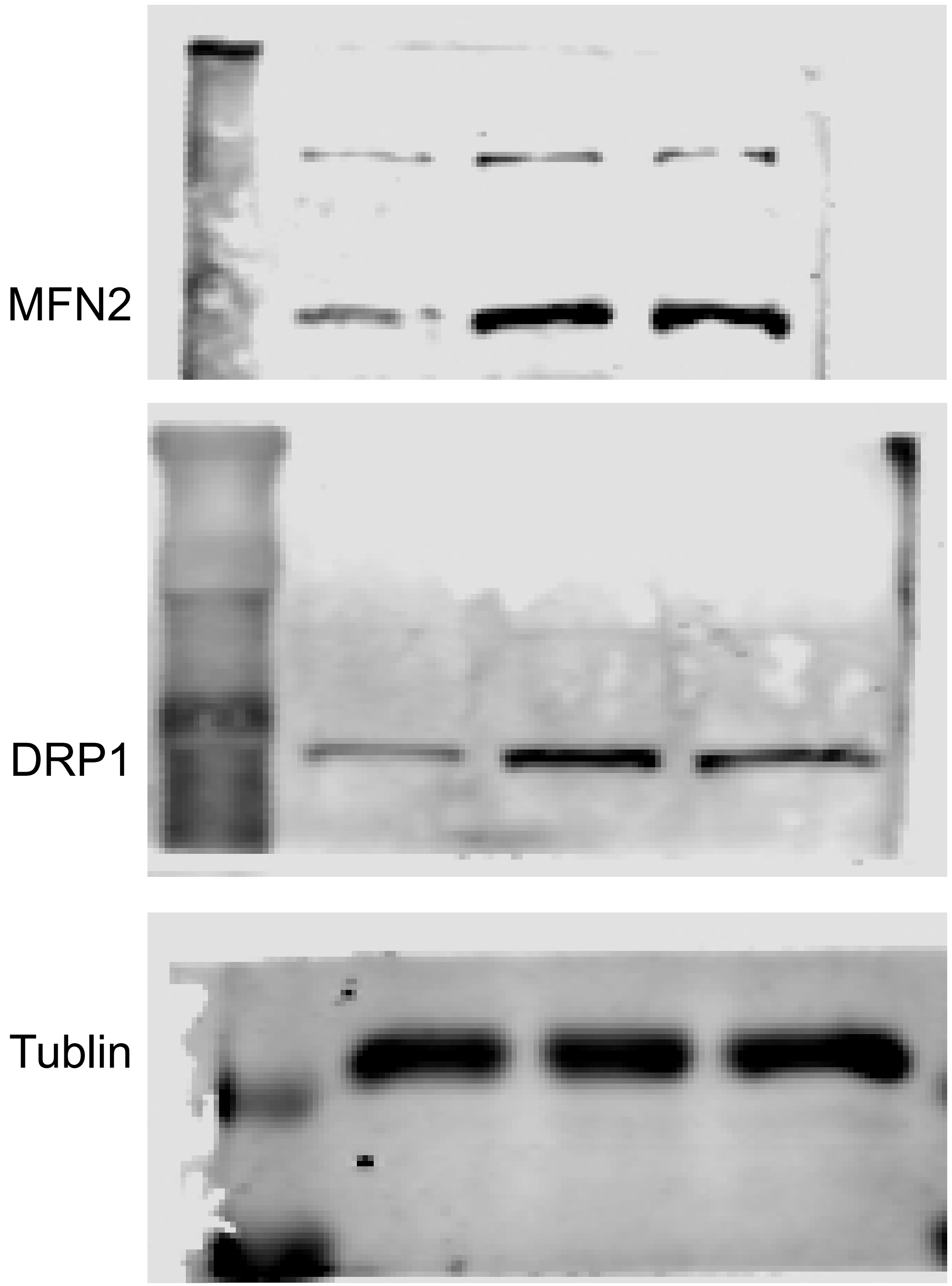

Figure 4j

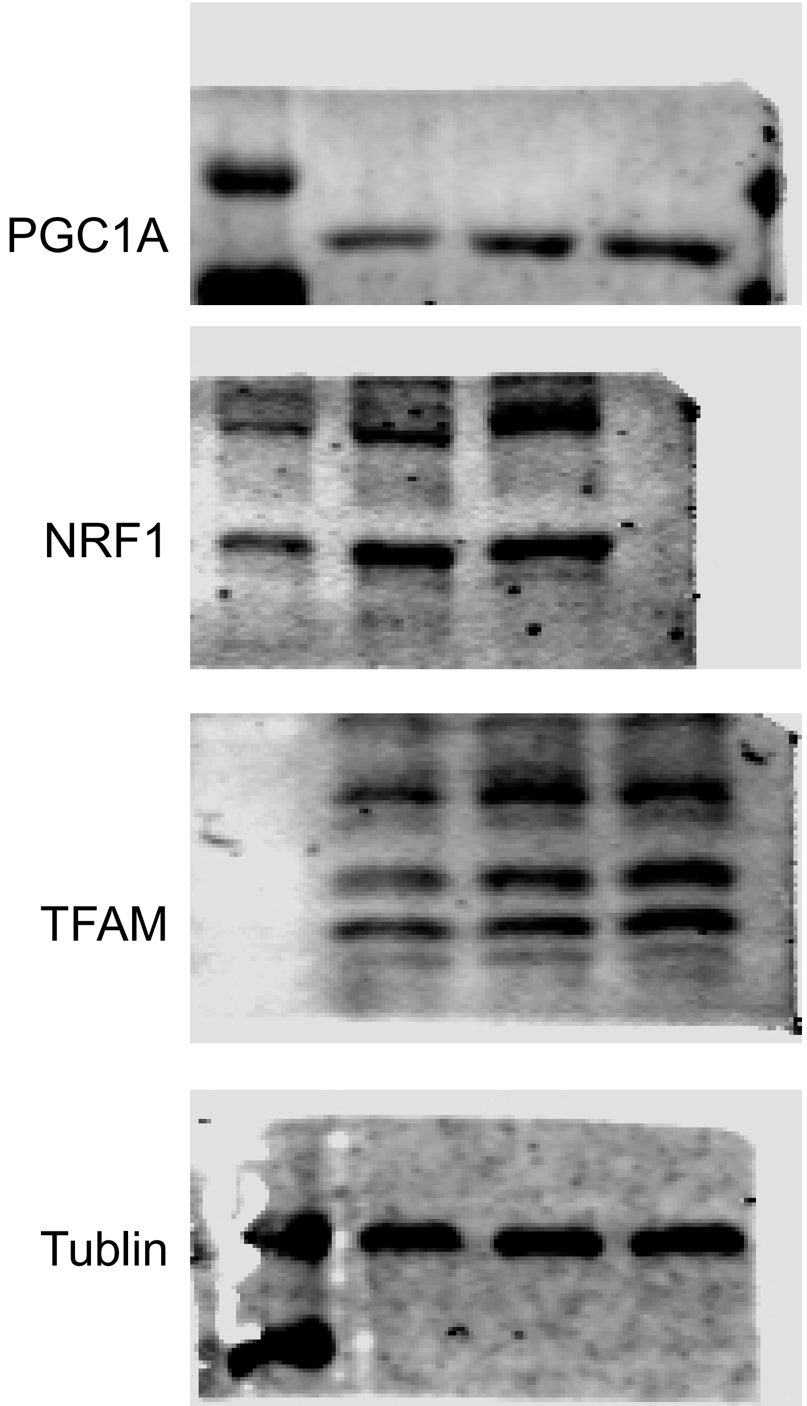

Figure 5c

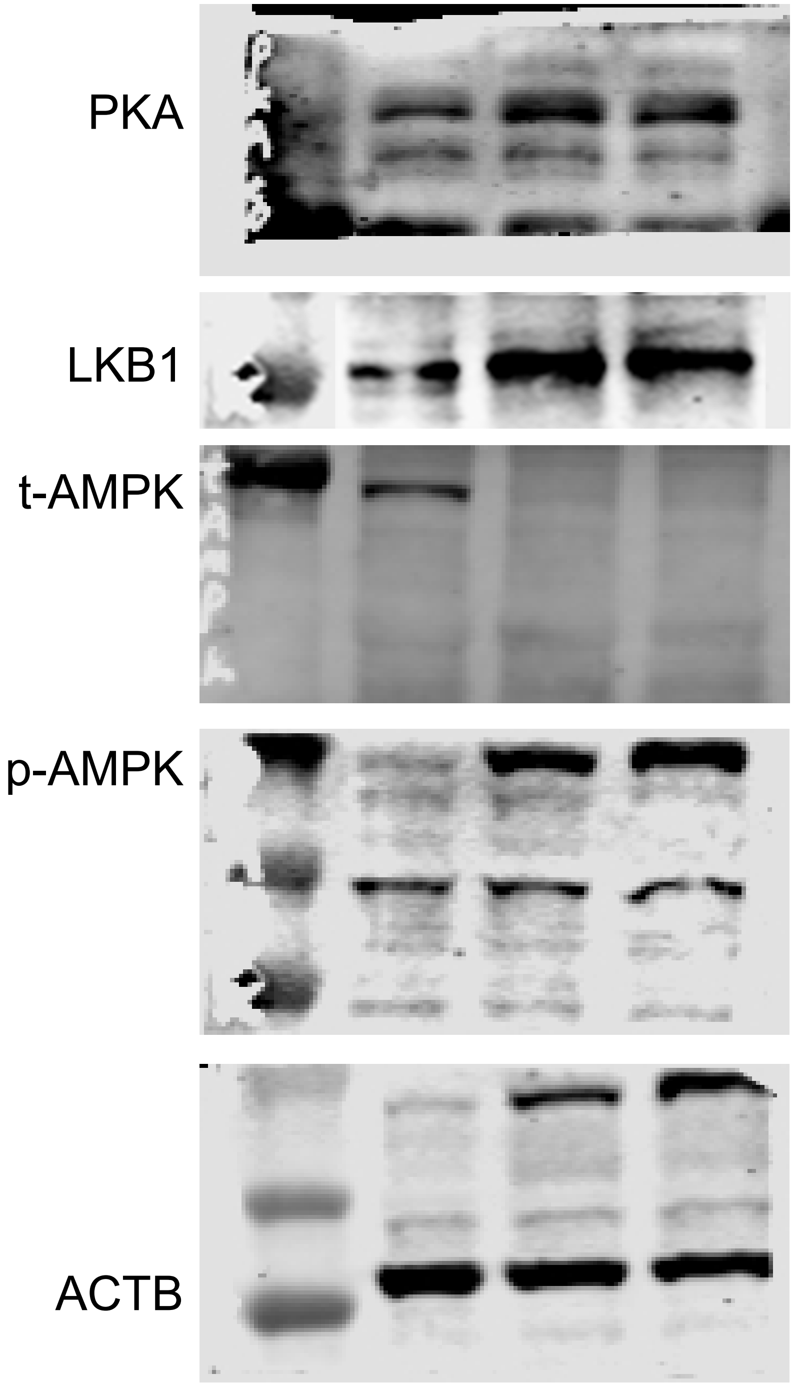

Figure 5d

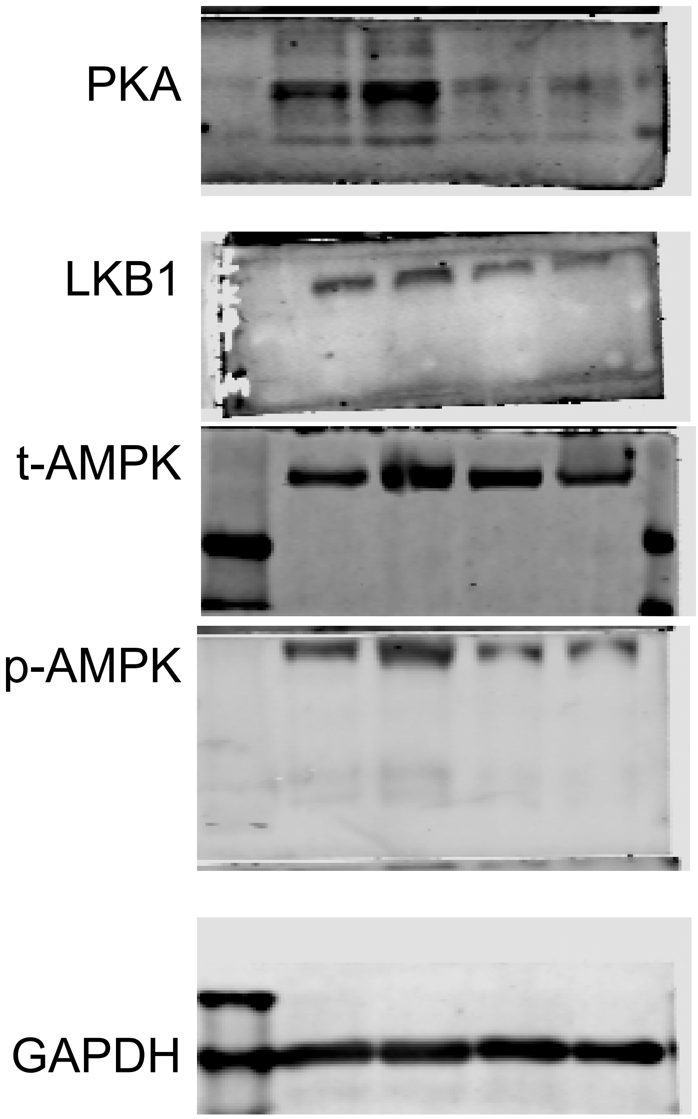

Figure 5e

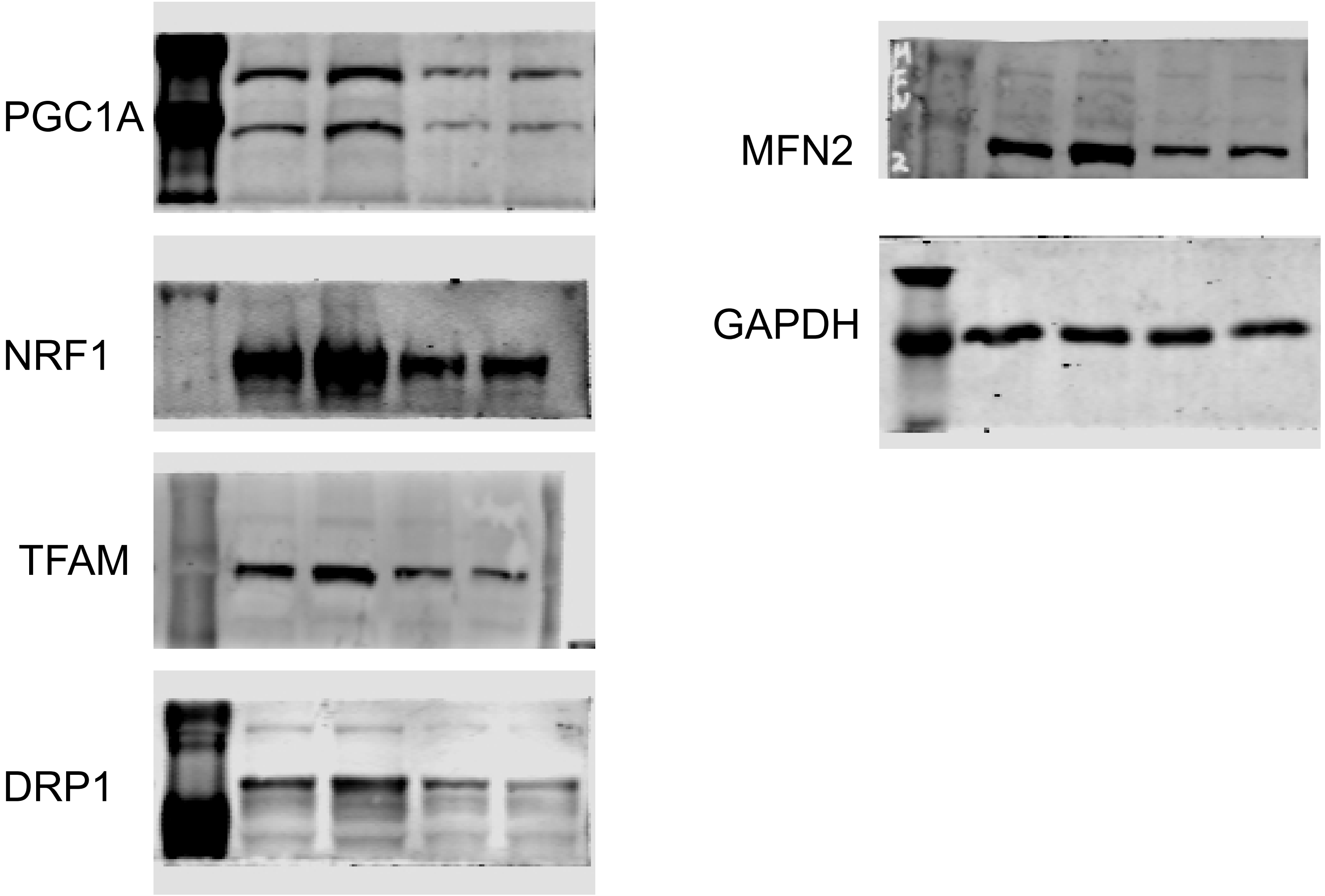

Figure 6d

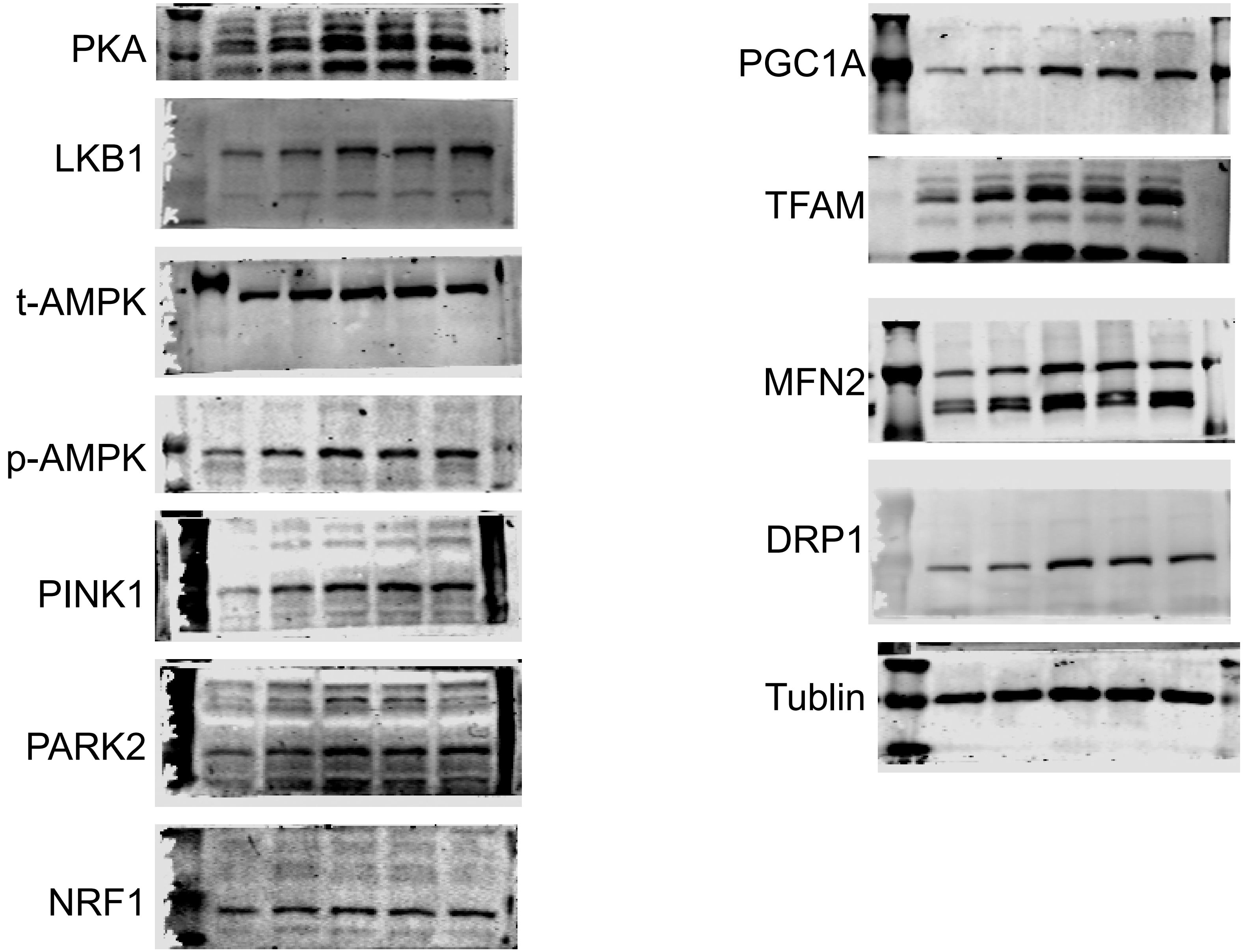
PKA
LKB1
t-AMPK
p-AMPK
PINK1
PARK2
NRF1
PGC1A
TFAM
MFN2
DRP1
Tublin

Figure 6e

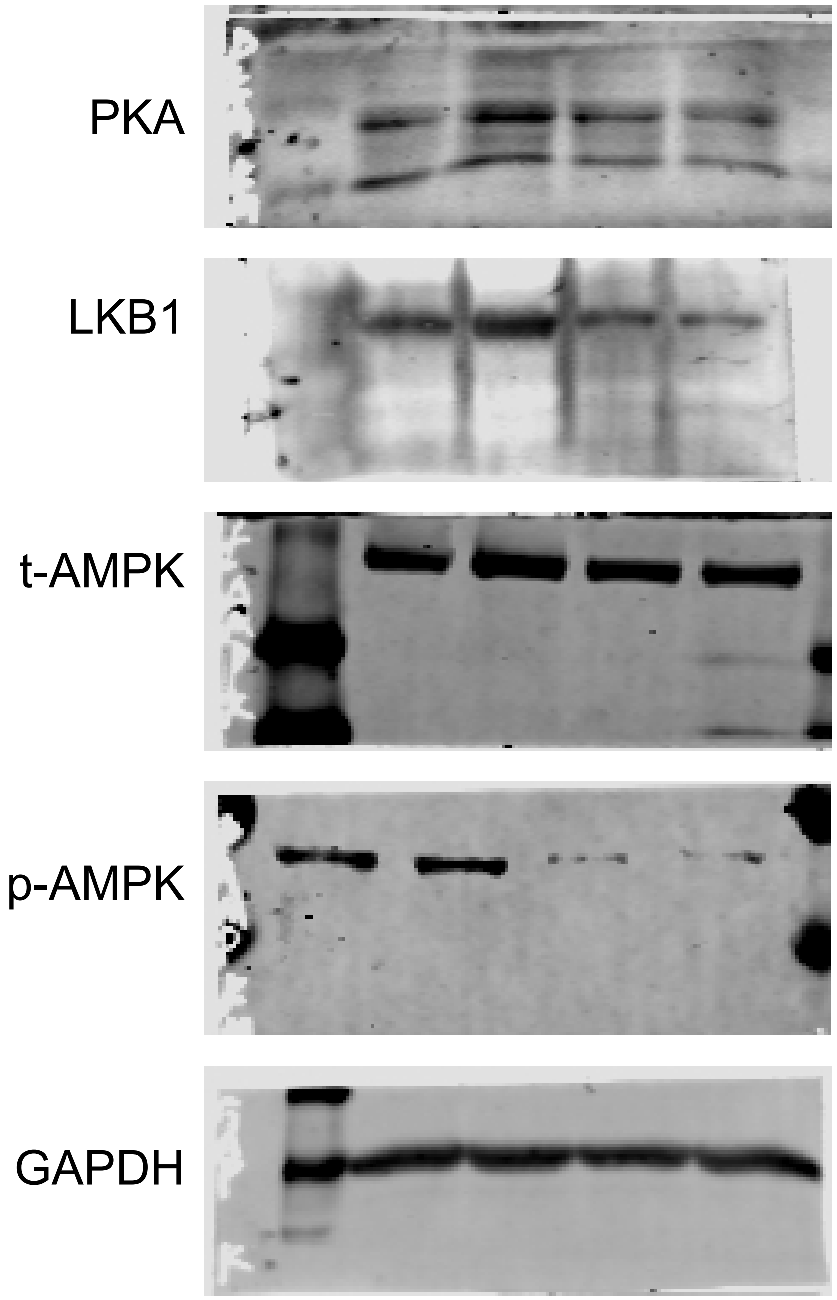

Figure 6g

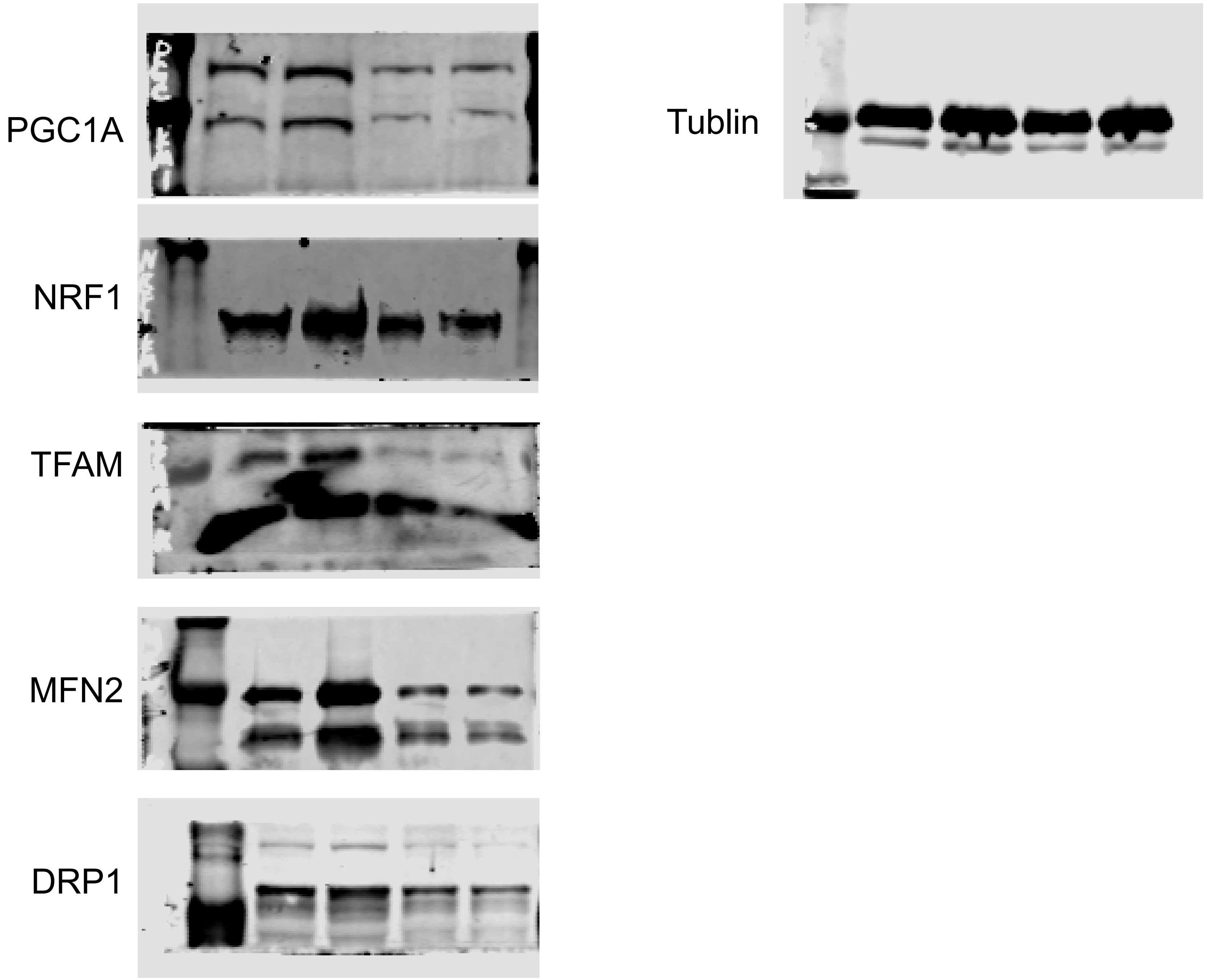

Figure 8a

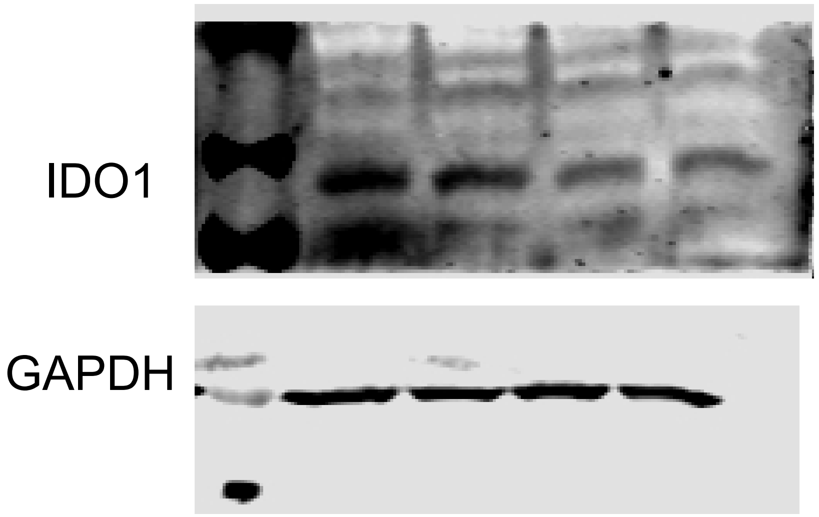

Figure S4a

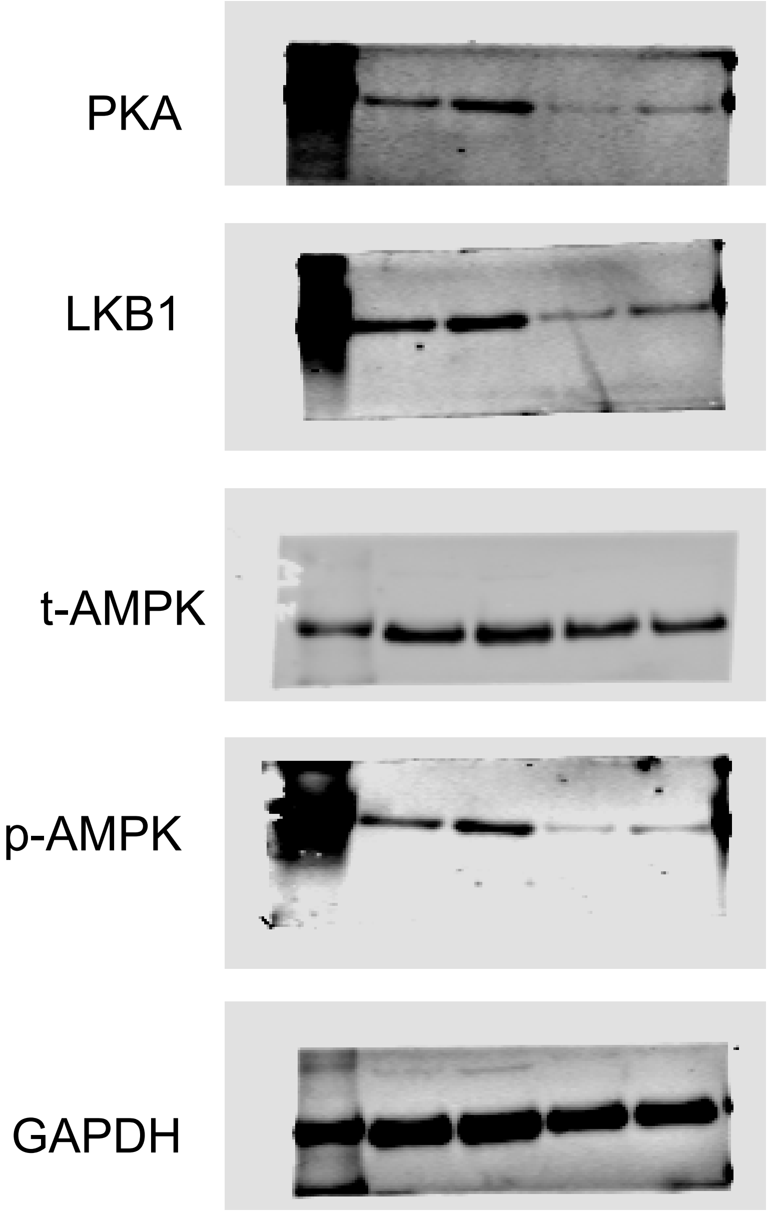

Figure S4b

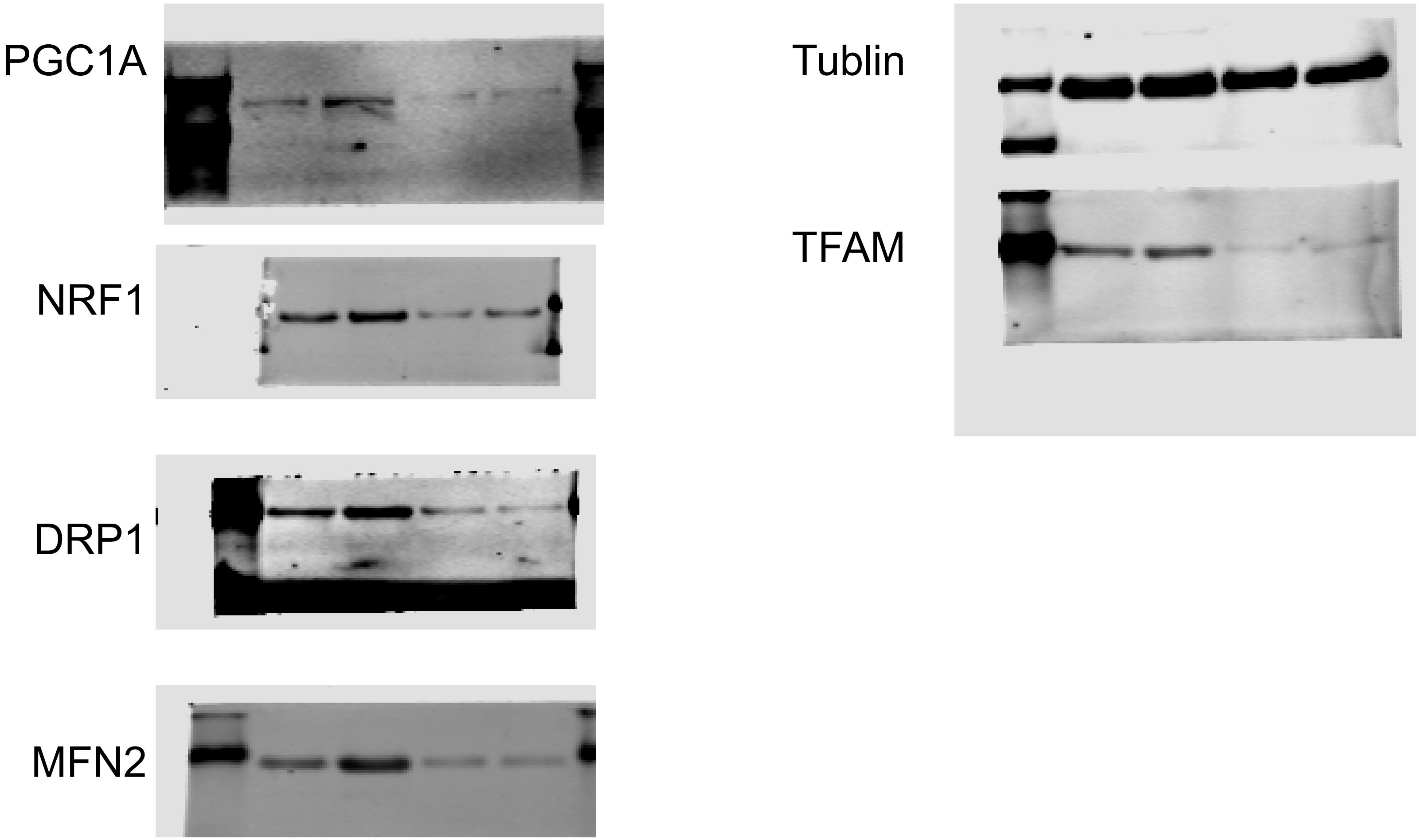

Figure S6a

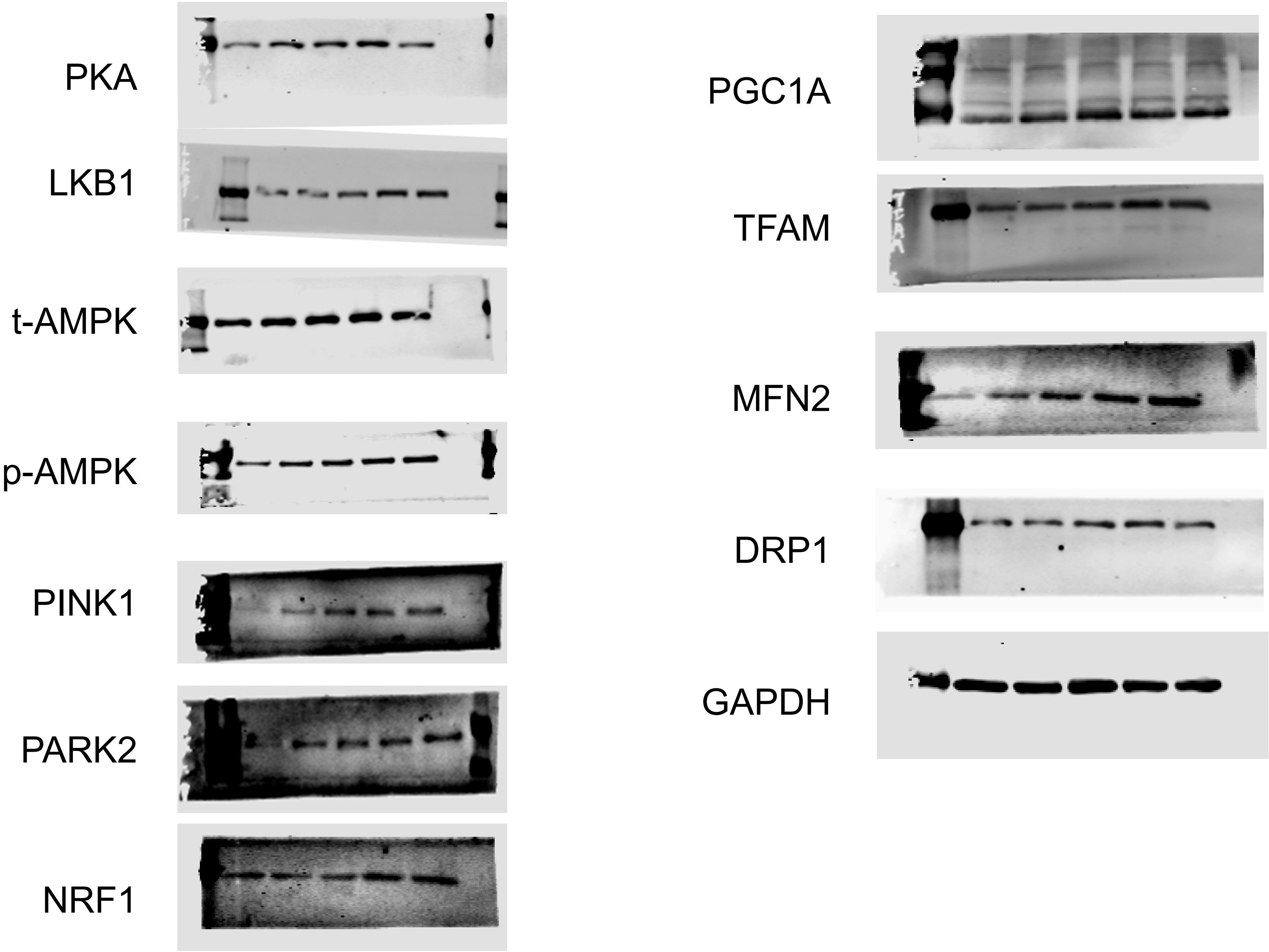
PKA
LKB1
t-AMPK
p-AMPK
PINK1
PARK2
NRF1
PGC1A
TFAM
MFN2
DRP1
GAPDH

Figure S6b

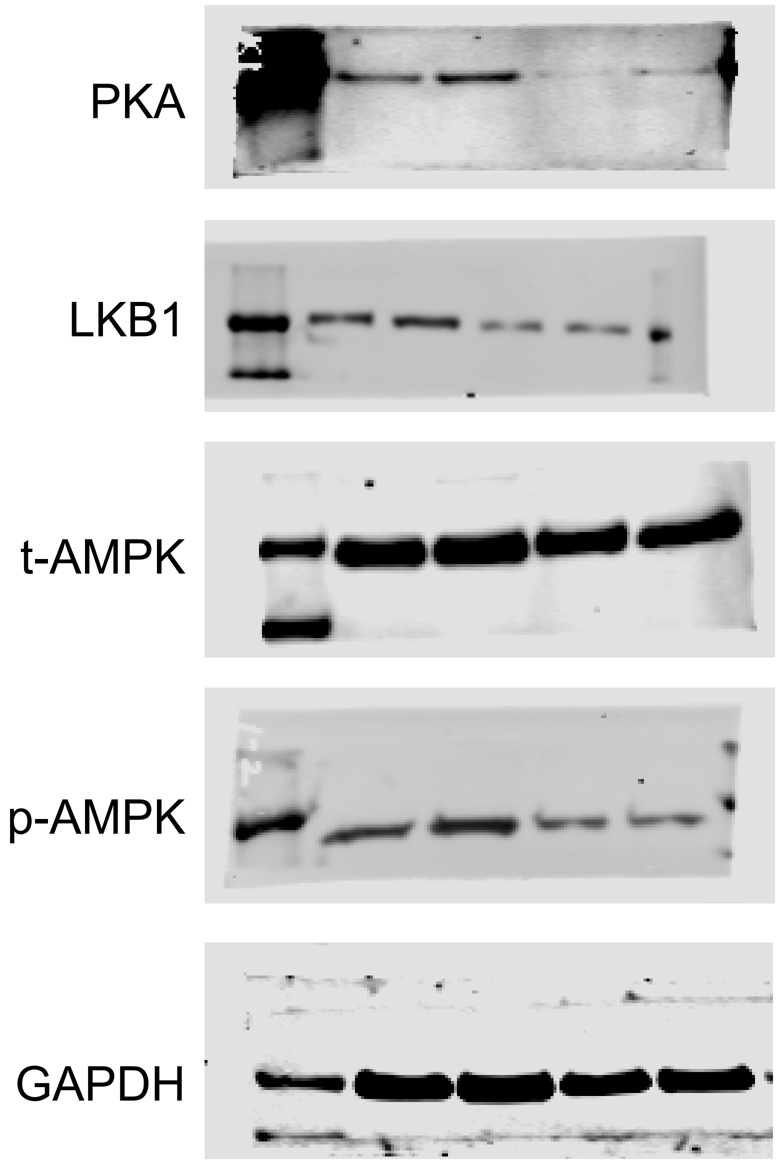

Figure S6c

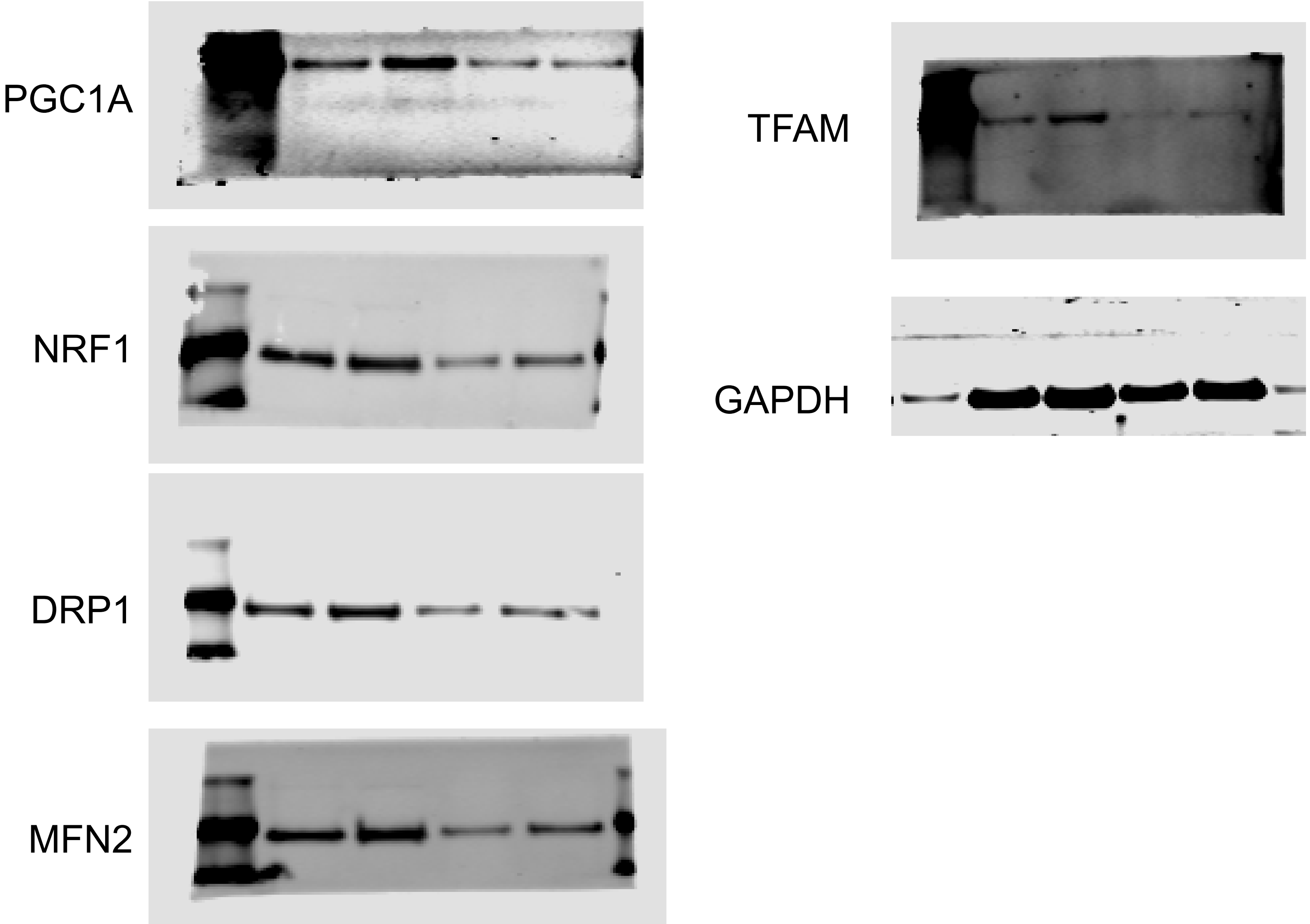

Figure S8a

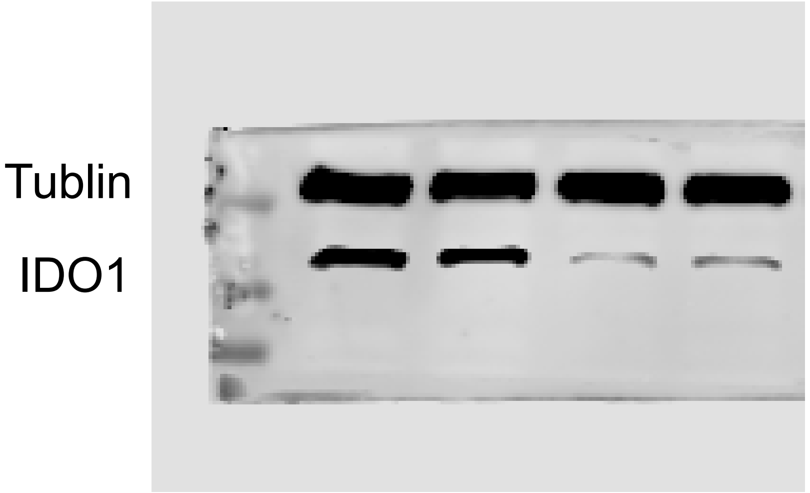

Supplement: Supplementary file 7 — Supplementary material-Original image [file 41419_2024_7068_MOESM7_ESM.pdf]
